# Synthesis, Cytotoxicity and Antioxidant Activity Evaluation of Some Thiazolyl–Catechol Compounds

**DOI:** 10.3390/antiox13080937

**Published:** 2024-08-01

**Authors:** Alexandra Cătălina Cornea, Gabriel Marc, Ioana Ionuț, Cristina Moldovan, Ionel Fizeșan, Andreea-Elena Petru, Ionuț-Valentin Creștin, Adrian Pîrnău, Laurian Vlase, Ovidiu Oniga

**Affiliations:** 1 Department of Pharmaceutical Chemistry, “Iuliu Hațieganu” University of Medicine and Pharmacy, 41 Victor Babeș Street, 400012 Cluj-Napoca, Romania; alexandra.cata.cornea@elearn.umfcluj.ro (A.C.C.); ionut.ioana@umfcluj.ro (I.I.); cmoldovan@umfcluj.ro (C.M.); ooniga@umfcluj.ro (O.O.); 2 Department of Toxicology, Faculty of Pharmacy, “Iuliu Hațieganu” University of Medicine and Pharmacy, 8 Victor Babeș, 400012 Cluj-Napoca, Romania; andreea.elen.petru@elearn.umfcluj.ro (A.-E.P.); ionut.vale.crestin@elearn.umfcluj.ro (I.-V.C.); 3 National Institute for Research and Development of Isotopic and Molecular Technologies, 67-103 Donath Street, 400293 Cluj-Napoca, Romania; adrian.pirnau@itim-cj.ro; 4 Department of Pharmaceutical Technology and Biopharmaceutics, “Iuliu Hațieganu” University of Medicine and Pharmacy, 41 Victor Babeș Street, 400012 Cluj-Napoca, Romania; laurian.vlase@umfcluj.ro

**Keywords:** thiazole, catechol derivatives, DFT, antioxidant, cytotoxic

## Abstract

A series of thiazolyl–catechol compounds with antioxidant and cytotoxic activities were synthesized by a Hantzsch heterocyclization, using diverse thioamides as the thiocarbonyl component and 4-chloroacetyl-catechol as haloketone. These compounds were characterized by MS, IR spectroscopy, and NMR. Their antioxidant potential was evaluated by antiradical, electron transfer, and ferrous ion chelation assays using ascorbic acid, Trolox, and EDTA-Na_2_ as references. The cytotoxicity of the synthesized compounds was evaluated on two different cell types, normal human foreskin fibroblasts (BJ) and human pulmonary malignant cells (A549), using gefitinib as a reference anticancer drug. The results obtained from the tests highlighted compounds **3g** and **3h** with significant antioxidant activities. The highest cytotoxic potency against A549 cells was exhibited by compounds **3i** and **3j**, while compound **3g** demonstrated exceptional selectivity on malignant cells compared to gefitinib. These promising results encourage further investigation into targeted modifications on position 2 of the thiazole ring, in order to develop novel therapeutic agents.

## 1. Introduction 

The oxidation process in human organisms is a fundamental biochemical reaction essential for maintaining cellular homeostasis and supporting life. This process involves the transfer of electrons from one molecule to another, which occurs predominantly within the mitochondria during cellular respiration [1,2]. At this level, organic molecules such as glucose are oxidized to generate energy in the form of adenosine triphosphate (ATP), which is vital for powering various cellular activities. Therefore, while oxidation is indispensable for energy production and metabolic functions, the balance between reactive oxygen species (ROS) generation and antioxidant mechanisms is critical to prevent cellular damage and maintain physiological integrity. This delicate equilibrium underscores the importance of the oxidation process in both sustaining life and protecting against pathological conditions [1,3,4].

Oxidative stress, which is characterized by an imbalance between ROS production and the body’s antioxidant defense mechanisms, plays an important role in the pathogenesis and progression of human cancers. ROS, including superoxide anions, hydrogen peroxide, and hydroxyl radicals, are generated through metabolic processes such as mitochondrial respiration and NADPH oxidase activity. Under physiological conditions, ROS levels are tightly regulated by antioxidant enzymes like superoxide dismutase, catalase, and glutathione peroxidase. When ROS production exceeds the capacity of these defenses, oxidative stress ensues, leading to oxidative damage to DNA, proteins, and lipids. This damage can result in mutations, genomic instability, and alterations in cellular functions, thereby contributing to carcinogenesis by promoting oncogene activation, tumor suppressor gene inactivation, and modifications in signaling pathways that regulate cell proliferation, apoptosis, and metastasis [3,5,6,7].

Oxidative stress and inflammation are intricately connected processes that play significant roles in the pathophysiology of various diseases. ROS generated during oxidative stress can activate key inflammatory signaling pathways, including NF-κB and AP-1, leading to the upregulation of pro-inflammatory cytokines and adhesion molecules. This interaction creates a vicious cycle where inflammation further enhances ROS production, exacerbating oxidative stress and perpetuating tissue damage. This correlation is critical in the progression of chronic inflammatory conditions, like atherosclerosis, rheumatoid arthritis, and certain cancers, where sustained oxidative stress and inflammation contribute to the disease pathology and progression [2,8,9,10].

The thiazole ring, an aromatic five-membered heterocycle containing both sulfur and nitrogen atoms, is a fundamental scaffold in medicinal chemistry due to its presence in a variety of biologically active compounds. Thiazole rings are found in several naturally occurring molecules and synthetic drugs, such as the anticancer agent dasatinib, which is used for treating chronic myeloid leukemia and acute lymphoblastic leukemia. The versatility of the thiazole ring allows it to participate in diverse pharmacological roles, including antimicrobial, anti-inflammatory, and anticancer activities. For instance, thiazole derivatives have been investigated for their potential to inhibit key enzymes involved in oxidative stress, such as NADPH oxidase, and to suppress inflammatory mediators, thereby offering therapeutic benefits in conditions characterized by chronic inflammation and oxidative damage, such as rheumatoid arthritis and cancer. The integration of thiazole-based compounds into drug design exemplifies the strategic approach to developing new therapies that target the interconnected pathways of oxidative stress and inflammation [11,12,13].

Polyphenols, a diverse group of natural and synthetic compounds, are pivotal in the fields of antioxidant and anticancer research due to their ability to modulate oxidative stress and inhibit carcinogenesis. Natural polyphenols, found in fruits, vegetables, tea, and wine, include flavonoids, phenolic acids, and stilbenes. These compounds such as resveratrol, curcumin, and epigallocatechin gallate have shown significant potential in scavenging ROS, thereby reducing oxidative stress and mitigating inflammation, which are key contributors to cancer development. Synthetic polyphenols, designed to enhance bioavailability and efficacy, also demonstrate strong antioxidant properties. These compounds exert anticancer activity by inducing apoptosis, inhibition of cell proliferation, and modulation of key signaling pathways like inhibition of cell proliferation, induction of apoptosis, and suppression of metastasis [14,15,16].

Based on the well-documented versatility of the thiazole and polyphenol structural fragments, we decided to synthesize a series of hybrid compounds with a thiazolyl–catechol structure. Inspired by our group’s previous research results, which indicated that thiazolyl–phenolic compounds exhibit significant antioxidant and cytotoxic activities, we decided to develop a new series of such compounds [17]. 

Focused on the desired outcome, we chose different thioamides with both electron-donating and electron-accepting effects to evaluate the influence of the substituent in position 2 of the thiazole ring on the antioxidant and anticancer activities. These compounds were synthesized through chemical methods using the Hantzsch reaction [12]. Their antioxidant activities were evaluated through a series of seven tests, and their cytotoxic effects were assessed on two cell lines: a healthy cell line (BJ) and a cancerous cell line (A549).

## 2. Materials and Methods

### 2.1. Chemicals, Consumables and Instruments

#### 2.1.1. Chemicals and Consumables

All the chemicals used for synthesis, purification, structural analysis, and evaluation of the biological activity had an appropriate grade of purity, according to the needs of each step, and they were all purchased from the local authorized suppliers.

The thioamides **1a–e** and **1g–h** used for the synthesis of compounds **3a–e** and **3g–h**, respectively, were produced by Sigma-Aldrich or Merck (Merck KGaA, Darmstadt, Germany), Alfa Aesar (Thermo Scientific, Waltham, MA, USA), or TCI (Tokyo Chemical Industry UK Ltd., Oxford, UK). On the other hand, the thioamides **1f** and **1i–j** used for the synthesis of compounds **3f** and **3i–j**, respectively, were previously obtained through synthesis by our group and were previously reported and characterized [18,19,20]. Their obtention is presented in Scheme S1 in the Supplementary Materials.

For the preparation of the solutions of compounds **3a–j**, dimethyl sulfoxide (DMSO) (≥99%) was purchased from Merck (Merck KGaA, Darmstadt, Germany). 

For the cytotoxicity assays, we used Dulbecco’s modified Eagle’s medium (DMEM) with low glucose or with high glucose, fetal bovine serum (FBS), Penicillin/Streptomycin, Phosphate-Buffered Saline (PBS), and Trypsin-EDTA from Gibco (Thermo Scientific, Paisley, UK). The cell lines used as models in the present study to evaluate the cytotoxicity of the synthesized compounds were normal human foreskin fibroblasts (BJ) and human pulmonary malignant cells (A549). Both cell lines were purchased from ATCC (Manassas, VA, USA).

#### 2.1.2. Instruments

For the physicochemical and spectral characterization of the obtained compounds, we measured the melting point, mass spectra, IR spectra, and NMR spectra. The melting point measurement was made using a melting point MPM-H1 device (Schorpp Gerätetechnik, Überlingen, Germany) by the glass capillary method. For all compounds, the mass spectra were recorded using an Agilent 1100 series device connected to an Agilent Ion Trap SL mass spectrometer (Agilent Technologies, Santa Clara, CA, USA) in negative ionization mode. Using an FT/IR 6100 spectrometer (Jasco, Cremella, Italy), we recorded the IR spectra for the compounds, after their compression in KBr pellets under vacuum. The NMR spectra (^1^H-NMR and ^13^C-NMR) were recorded using an Avance NMR spectrometer (Bruker, Karlsruhe, Germany) after dissolution of the powders of the compounds in dimethyl sulfoxide-*d*_6_ (DMSO-*d*_6_). The NMR spectrometer was calibrated with tetramethylsilane and the signals were referred to the residual solvent peak. The peaks in the NMR spectra were found in the expected region, with expected intensity and multiplicity, and were assigned to the structural moieties of the compounds, according to their multiplicity (*s*—singlet; *d*—doublet; *dd*—doublet of doublets; *m*—multiplet). To indicate the location of hydrogen or carbon atoms within a specific region of the molecule, certain abbreviations were used: ctc—catechol fragment from position 4 of the thiazole; Ar—for all aromatic fragments in position 2 of the thiazole; Th—thiazole; Py—pyridine).

To perform the antiradical, electron transfer, and ferrous ion chelation, the absorbance of the resulting mixtures was spectrophotometrically assessed using a Specord 210 PLUS UV-Vis device (Analytik Jena AG, Jena, Germany) in low-volume single-use 10 mm plastic cuvettes.

The DFT calculations were performed on a machine with an Intel Core i7-12700KF running Windows 10 (Microsoft. Redmond, WA, USA).

In the cytotoxicity assay, a Synergy 2 Multi-Mode Microplate Reader (BioTek, Winooski, VT, USA) was used to measure the fluorescence of the samples.

#### 2.1.3. Synthesis Protocol and Characterization of Final Compounds

**Scheme 1:**
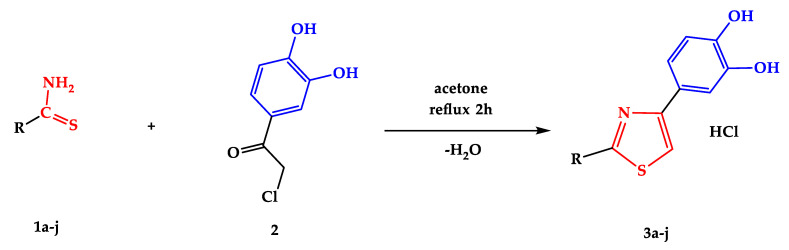
Synthesis of compounds **3a–j** through Hantzsch heterocyclization, starting from thioamides **1a–j** and 4-chloroacetyl-catechol (**2**). Thioamides **1f**, **1i**, and **1j** were reported in our group’s previous papers [18,19,20]. Their obtention is presented in Scheme S1 in the Supplementary Materials. The description of the R fragment is presented in Table 1.

For the synthesis of final compounds **3a–j**, 2 mmol of thioamides (**1a–j**) was put into a glass flask with 10 mL of anhydrous acetone and 2 mmol of 4-chloroacetyl-catechol (**2**) as illustrated in Scheme 1. The mixture had to react for approximately two hours under a condenser in a heating mantle with magnetic stirring. The evolution of the reaction was monitored by thin-layer chromatography (TLC) on silica gel 60 GF_254_ plastic sheets (Merck KGaA, Darmstadt, Germany). When the reaction was completed, the resulting precipitate was filtered under a vacuum when the mixture was still hot to prevent the impurification of the precipitate with insoluble impurities in the cold [12,21]. All compounds were isolated as hydrochlorides to enhance their long-term storage stability and facilitate their solubilization in the solvents used for the antioxidant and cytotoxic assays.

All compounds were analyzed using consecrated spectral methods: MS (Mass Spectrometry), IR (Infrared Spectroscopy), and NMR (Nuclear Magnetic Resonance). The structural analysis was performed only for the newly synthesized compounds because compounds **3a–c** were already reported and characterized by other authors [22,23,24,25].

4-(2-(4-bromophenyl)thiazol-4-yl)benzene-1,2-diol (**3d**): yellow solid; mp = 220 °C; yield = 85%; FT IR (KBr) v_max_ cm^−1^: 613 (c-Br); 3130, 3553 (phenolic OH); MS: *m*/*z* = 347.7; ^1^H-NMR (DMSO-*d*_6_, 500 MHz) δ: 6.83 (*d*, 1H, ctc, *J* = 8 Hz), 7.32 (*dd*, 1H, ctc, *J*_1_ = 2 Hz, *J*_2_ = 8 Hz), 7.47 (*d*, 1H, ctc, *J* = 2.5 Hz), 7.71 (*d*, 1H, Ar, *J* = 8.5 Hz), 7.86 (*s*, 1H, Th), 7.92 (*d*, 1H, Ar, *J* = 8.5 Hz); ^13^C-NMR (DMSO-*d*_6_, 125 MHz) δ: 112.2 (Th-C5), 113.9 (ctc), 115.9 (ctc), 117.6 (ctc), 123.4 (Ar), 125.6 (ctc), 127.9 (Ar), 132.2 (Ar), 132.3 (Ar), 145.4 (ctc-OH), 145.9 (ctc-OH), 155.9 (Th-C4), 165.0 (Th-C2).

4-(2-(m-tolyl)thiazol-4-yl)benzene-1,2-diol (**3e**): yellow solid; mp = 196 °C; yield = 87%; FT IR (KBr) v_max_ cm^−1^: 3109, 3426 (phenolic OH); MS: *m*/*z* = 282.1; ^1^H-NMR (DMSO-*d*_6_, 500 MHz) δ: 2.40 (*s*, 3H, -CH_3_), 6.83 (*d*, 1H, ctc, *J* = 8 Hz), 7.29–7.33 (*m*, 2H, Ar+ctc), 7.40 (*t*, 1H, Ar, *J* = 8 Hz), 7.48 (*d*, 1H, ctc, *J* = 2 Hz), 7.77 (*d*, 1H, Ar, *J* = 8 Hz), 7.81 (*s*, 1H, Th), 7.81 (*s*, 1H, Ar); ^13^C-NMR (DMSO-*d*_6_, 125 MHz) δ: 20.9 (-CH_3_), 111.6 (Th-C5), 113.9 (ctc), 115.9 (ctc), 117.6 (ctc), 123.3 (1C, Ar), 125.7 (ctc), 126.4 (Ar), 129.1 (Ar), 130.8 (Ar), 133.1 (Ar), 138.6 (Ar), 145.4 (ctc-OH), 145.8 (ctc-OH), 155.6 (Th-C4), 166.4 (Th-C2).

4-(2-(3,4,5-trimethoxyphenyl)thiazol-4-yl)benzene-1,2-diol (**3f**): yellow solid; mp = 219 °C; yield = 72%; FT IR (KBr) v_max_ cm^−1^: 3556, 3047 (phenolic OH), 1265, 1021 (-OCH_3_), 1252 (-OCH_3_), 1080 (-OCH_3_); MS: *m*/*z* = 359.5; ^1^H-NMR (DMSO-*d*_6_, 500 MHz) δ: 3.73 (*s*, 3H, -CH_3_), 3.89 (*s*, 6H, -CH_3_), 6.83 (*d*, 1H, ctc, *J* = 8 Hz), 7.24 (*s*, 2H, Ar), 7.32 (*dd*, 1H, ctc, *J*_1_ = 2 Hz, *J*_2_ = 8 Hz), 7.46 (*d*, 1H, ctc, *J* = 2Hz), 7.79 (*s*, 1H, Th-C_5_); ^13^C-NMR (DMSO-*d*_6_, 125 MHz) δ: 56.1 (-OCH_3_), 60.2 (-OCH_3_), 103.5 (C, Ar), 111.6 (Th-C_5_), 113.9 (ctc), 115.9 (ctc), 117.7 (ctc), 125.7 (ctc), 128.8 (Ar), 139.3 (Ar), 145.4 (ctc-OH), 145.8 (ctc-OH), 153.3 (C, Ar), 155.5 (Th-C_4_), 166.2 (Th-C_2_).

4-(2-(pyridin-4-yl)thiazol-4-yl)benzene-1,2-diol (**3g**): orange solid; mp = 240 °C; yield = 20%; FT IR (KBr) v_max_ cm^−1^: 3052, 3549 (phenolic OH); MS: *m*/*z* = 269.1; ^1^H-NMR (DMSO-*d*_6_, 500 MHz) δ: 6.86 (*d*, 1H, ctc, *J* = 8.5 Hz), 7.37 (*dd*, 1H, ctc, *J*_1_ = 2 Hz, *J*_2_ = 8 Hz), 7.50 (*d*, 1H, ctc, *J* = 2.5 Hz), 8.25 (*s*, 1H, Th), 8.39 (*d*, 2H, Py, *J* = 3.5 Hz), 8.94 (*d*, 2H, Py, *J* = 6.5 Hz); ^13^C-NMR (DMSO-*d*_6_, 125 MHz) δ: 113.9 (Th-C5), 114.9 (ctc), 115.9 (ctc), 117.8 (ctc), 122.0 (Py), 124.5 (ctc), 145.5 (ctc-OH), 145.6 (ctc-OH), 146.6 (Py), 153.5 (Py), 157.3 (Th-C4), 161.2 (Th-C2).

4-(2-(pyridin-2-yl)thiazol-4-yl)benzene-1,2-diol (**3h**): brown solid; mp = 230 °C; yield = 20%; FT IR (KBr) v_max_ cm^−1^: 3109, 3550 (phenolic OH); MS: *m*/*z* = 269.1; ^1^H-NMR (DMSO-*d*_6_, 500 MHz) δ: 6.83 (*d*, 1H, ctc, *J* = 8 Hz), 7.33 (*dd*, 1H, ctc, *J_1_* = 1.5 Hz, *J_2_* = 8.5 Hz), 7.47 (*d*, 1H, ctc, *J* = 2.5 Hz), 7.50–7.52 (*m*, 1H, Py), 7.93 (*s*, 1H, Th), 7.99–8.02 (*m*, 1H, Py), 8.20 (*d*, 1H, Py, *J* = 7.5 Hz), 8.644–8.653 (m, 1H, Py); ^13^C-NMR (DMSO-*d*_6_, 125 MHz) δ: 113.7 (Th-C5), 114.1 (ctc), 115.8 (ctc), 117.5 (ctc), 119.1 (Py), 125.0 (ctc), 125.7 (Py), 137.8 (Py), 145.4 (ctc-OH), 145.8 (ctc-OH), 149.6 (Py), 150.4 (Py), 156.1 (Th-C4), 167.5 (Th-C2). 

4-(2′-phenyl-[2,4′-bithiazol]-4-yl)benzene-1,2-diol (**3i**): yellow solid; mp = 258 °C; yield = 40%; FT IR (KBr) v_max_ cm^−1^: 3045, 3259 (phenolic OH); MS: *m*/*z* = 351.7; ^1^H-NMR (DMSO-*d*_6_, 500MHz) δ: 6.83 (*d*, 1H, ctc, *J* = 8 Hz), 7.33 (*dd*, 1H, ctc, *J*_1_ = 2 Hz, *J*_2_ = 8 Hz), 7.47 (*d*, 1H, ctc, *J* = 2 Hz), 7.56 (*m*, 3H, Ar), 7.84 (*s*, 1H, Th1), 8.03 (*m*, 2H, Ar), 8.32 (*s*, 1H, Th2); ^13^C-NMR (DMSO-*d*_6_, 125 MHz) δ: 112.3 (Th1-C5), 113.8 (ctc), 115.8 (ctc), 117.1 (ctc), 117.6 (Th2-C5), 125.6 (ctc), 126.3 (Ar), 129.3 (Ar), 130.8 (Ar), 132.2 (Ar), 145.4 (ctc-OH), 145.8 (ctc-OH), 149.3 (Th2-C4), 155.9 (Th-C4), 161.1 (Th-C2), 167.9 (Th2-C2).

4-(2′-(p-tolyl)-[2,4′-bithiazol]-4-yl)benzene-1,2-diol (**3j**): yellow solid, mp = 265 °C; yield = 41%; FT IR (KBr) v_max_ cm^−1^: 3036, 3288 (phenolic OH); MS: *m*/*z* = 367.1; ^1^H-NMR (DMSO-*d*_6_, 500MHz) δ: 2.38 (*s*, 3H, -CH_3_), 6.83 (*d*, 1H, ctc, *J* = 8.5 Hz), 7.32 (*dd*, 1H, ctc, *J*_1_ = 2.5 Hz, *J*_2_ = 8.5 Hz), 7.37 (*d*, 2H, Ar), 7.46 (*d*, 1H, ctc, *J* = 2.5 Hz), 7.86 (*s*, 1H, Th1), 7.91 (*d*, 2H, Ar), 8.27 (*s*, 1H, Th2); ^13^C-NMR (DMSO-*d*_6_, 125 MHz) δ: 21.0 (-CH3), 112.3 (Th-C5), 113.8 (ctc), 115.8 (ctc), 116.6 (Th2-C5), 117.6 (ctc), 125.6 (ctc), 126.2 (Ar), 129.7 (Ar), 129.9 (Ar), 140.7 (Ar), 145.4 (ctc-OH), 145.8 (ctc-OH), 149.1 (Th2-C4), 155.9 (Th-C4), 161.1 (Th-C2), 168.1 (Th2-C2). 

### 2.2. Antiradical, Electron Transfer, and Ferrous Ion Chelation Assays

The stock solutions of the reference antioxidants (ascorbic acid, Trolox, EDTA-Na_2_) and compounds **3a–j** were prepared by dissolving the solid powders in DMSO, obtaining 2 mM stock solutions. Following this, an additional set of solutions (0.2 mM) for each compound was obtained by dilution with DMSO. Absorption spectra of the tested compounds covering the range from 400 nm to 800 nm were recorded, indicating no absorption peaks near the wavelengths employed in the experiments. All measurements in the presented assays were made against blank samples. To quantify the activity of the compounds **3a–j**, for the antiradical assays (DPPH and ABTS) and ferrous ion chelation, IC_50_ was calculated using Equation (1), while for the electron transfer assays (TAC, RP, FRAP and CUPRAC), the reference number of mole equivalent activity for each compound was calculated using Equation (2).

(1) $$radical\;scavenging\;(\%)=\frac{control\;absorbance-sample\;absorbance}{control\;absorbance}\times 100$$

(2) $$equivalents\;of\;control=\frac{sample\;absorbance}{control\;absorbance}$$

#### 2.2.1. Antiradical Assays

The DPPH˙ (2,2-diphenyl-1-picrylhydrazyl) radical-scavenging assay, initially reported by Brand-Williams et al., relies on transferring a hydrogen atom from the antioxidant substrate being analyzed to the stable violet radical of DPPH˙. This action converts DPPH˙ into a nonradical stable yellow compound. The reduction in absorbance of the DPPH˙ at λ = 517 nm corresponds to the quantity of neutralized reagent [26,27,28]. To perform the DPPH assay, we took 50, 75, 100, 125, and 150 µL of each sample from their 0.2 mM stock solutions, then we adjusted the volumes with DMSO to ensure all samples reached a uniform final volume. These solutions were mixed with 1 mL of DPPH reagent and incubated in the dark for 30 min, as presented in literature reports [17,29].

The decolorization assay of the green ABTS^•+^ (2,2′-azinobis-(3-ethylbenzothiazoline-6-sulfonic acid) to ABTS, based on the report of Re et al., was conducted following our group’s published report [17,30]. The chemical stability of the ABTS^•+^ reagent prior to use was checked at λ = 734 nm over one hour, ensuring a consistent absorption (approximately equal to 0.7). The reagent was prepared in potassium phosphate buffer (0.1 M, pH = 7.4) and activated overnight using MnO_2_ [17]. In all cuvettes, we added 100 μL of the tested compound solutions (0.2 mM) in increasing concentrations up to 200 µL and 2000 µL of the ABTS^•+^ reagent. The resulting mixtures were thoroughly shaken for 10 min at room temperature, in the absence of light. The optical absorbance of the resulting mixtures was then measured by spectrophotometry at λ = 734 nm [17,29]. 

#### 2.2.2. Electron Transfer Assays

The Total Antioxidant Capacity (TAC) assay was conducted following the methodology outlined in our previous publication, which was based on initial reports from the literature [17,29,31,32]. A volume of 10 mL of the reagent (0.6 M H_2_SO_4_, 28 mM Na_3_PO_4_ and 4 mM (NH_4_)_6_Mo_7_O_24_) and 1000 µL of the compound and standard solutions dispensed from the 2 mM stock solutions were mixed in sealed glass test tubes and incubated in a water bath at 95 °C for 1.5 h. After cooling the test tubes to room temperature, 1000 µL of each solution was diluted with 1000 µL of water. The absorbance of the solutions was then measured at λ = 695 nm.

In the reducing power (RP) assay, the tested compounds performed the reduction of ferric ions from potassium ferricyanide, forming ferrocyanide, which gives a blue complex. The protocol followed is an adaptation of previously reported methodologies [17,29]. In glass test tubes, 1000 µL of solutions containing compounds and standards measured from the 0.2 mM stock solutions were mixed with 400 µL of phosphate buffer (0.2 M, pH = 6.6) and 400 µL of K_3_[Fe(CN)_6_] solution (1% *w*/*v*). The tubes were sealed and incubated for 20 min in a water bath at 50 °C. After cooling to room temperature, 500 µL of trichloroacetic acid (10% *w*/*w*) was added to all test tubes The resulting mixtures were left for 0.5 h at room temperature. Subsequently, 250 µL of the resulting solutions were mixed with 140 µL of FeCl_3_ solution (0.1% *w*/*v*) and 1000 µL of distilled water, and the absorbance of the resulting solutions was determined at λ = 695 nm.

The Ferric-Reducing Antioxidant Potential (FRAP) assay relies on the transfer of an electron from the antioxidant being assessed to Fe^3+^, resulting in its conversion to Fe^2+^. The resulting Fe^2+^ ions are then chelated by 2,4,6-tripyridyl-s-triazine, giving a blue-colored complex, with an absorption maximum at λ = 593 nm [29,33]. A 500 µL amount of the solutions (0.2 mM) of compounds and reference compounds was mixed with 600 µL FRAP reagent and 1200 µL acetate buffer (0.3 M, pH = 3.6), according to previous reports [17,29]. The resulting solutions were mixed well in the dark, and their absorbance was measured spectrophotometrically.

The electron-donating capacity of compounds was assessed using the CUPRAC (CUPric-Reducing Antioxidant Capacity) method. The protocol for this assay is a modification of the reports of Alam et al., Özyürek et al., and Apak et al. [29,34,35]. Volumes of 250 µL of CuCl_2_ 10 mM, 1 mL of ammonium acetate buffer 1 M, and 250 µL of 7.5 mM neocuproine in ethanol were mixed with 125 µL of samples and reference compound solutions (0.2 mM). The mixtures were shaken for 30 min in the dark; after that, their absorbance was determined spectrophotometrically at λ = 450 nm.

#### 2.2.3. Ferrous Ion Chelation Assay

To evaluate the ability of compounds to chelate ferrous ions, an assay was performed using an adaptation of Dinis et al.’s initial report [36]. The solutions of the tested compounds (2 mM) were mixed well with 500 µL of FeSO_4_ (0.125 mM), and 500 µL of ferrozine 0.315 mM. After 10 min, the absorbance of the samples was determined spectrophotometrically at λ = 562 nm [29,37,38,39]. EDTA-Na_2_ was used as a positive control, due to its exceptional ion chelation properties.

### 2.3. In Silico Studies 

#### 2.3.1. Theoretical Quantum Calculations

For compounds **3a–j**, two main mechanisms were taken into account for their potential antioxidant mechanisms: one by electron release or acceptation, characterized by the frontier molecular orbital energy levels—Highest Occupied Molecular Orbital (HOMO) and Lowest Unoccupied Molecular Orbital (LUMO)—and the other by hydrogen atom transfer (HAT), by the heterolytic cleavage of the OH bond (Ar-OH → Ar-O• + H•). Single-electron transfer–proton transfer (SET-PT) and sequential proton loss electron transfer (SPLET) are considered in literature reports to be less possible for phenol molecules and were not evaluated in the current research. The calculation of O-H BDE was performed using BDE = H(ArO•) + H(H•) − H(ArOH) [40,41,42,43,44].

The calculations for the studied compounds were performed by Spartan20 1.1.2 (Wavefunction, Irvine, CA, USA) on the B3LYP level of theory with the 6-311++G(d,p) basis set for vacuum, non-polar solvent, and water to obtain evidence of how the solvent may influence the antioxidant potential of the studied molecules, by applying the Polarizable Continuum solvation model. The calculations and the resulting data provided were computed at 298.15K and 1 atm. The geometry optimization of the molecules was performed using the built-in Pulay Direct Inversion in the Iterative Subspace (DIIS) combined with geometric direct minimization [40,44,45]. For the compounds **3a–j**, we calculated the HOMO and LUMO frontier molecular orbitals, and derived descriptors such as HOMO-LUMO gap, hardness (η), and chemical potential (µ).

#### 2.3.2. Molecular Properties with Influence on the Pharmacokinetics

We used SwissADME to gather preliminary information on the molecular properties that could affect the pharmacokinetics of the compounds [46]. The assessed properties included the topological polar surface area (TPSA) [47], octanol–water partition coefficient (expressed as Moriguchi’s LogP) [48], water solubility, and any violations of Lipinski’s rule of five [49]. 

### 2.4. Cytotoxicity Studies 

BJ cells were maintained in Dulbecco’s modified Eagle’s medium (DMEM) with low glucose (1 g/L), while the A549 cells were seeded in DMEM with high glucose (5 g/L). All media were supplemented with 10% fetal bovine serum (FBS) and 1% Penicillin/Streptomycin. The cells were maintained in an incubator at 37 °C in a humidified atmosphere with 5% CO_2_ supplementation. Cellular media were refreshed every other day. Cells were either subcultured or used in experiments once they reached a confluency of 80–90%. 

The cytotoxicity of the synthesized compounds was evaluated by Alamar Blue (AB) assays performed on both cell lines, A549 and BJ, after a 48 h exposure, as previously reported [50,51]. The test uses the fluorometric redox indicator, resazurin, which is converted by metabolically active cells to resorufin, a fluorescent compound.

Briefly, approximately 7000 A549 cells and 4000 normal BJ cells were seeded in 100 µL cellular media in 96-well plates to achieve a confluency of 70–80% at the end of the experiment and left to attach overnight before exposure. Consequently, attached cells were washed with PBS and further exposed to the reference anticancer agent, gefitinib, and the synthesized compounds **3a–j** for 48 h at different concentrations, which were determined using the up-and-down method. Post-exposure, the media were removed, and the AB assay was performed, with cells being incubated with a 200 µM solution of resazurin for 4 h. The obtained fluorescence was measured at λ_excitation_ = 530/25; λ_emission_ = 590/35. 

The experiments included three biological replicates, each one including three technical replicates. The negative control (NC) consisted of cells exposed to a cell culture medium containing 0.2% DMSO and was used for data normalization (100%). 

For the potency assessment of the synthesized compounds, 50% inhibitory concentration (IC_50_) values were calculated for each cell line, from the dose–response curves obtained by fitting the experimental data with a 4-parameter logistic curve by using GraphPad Prism 6 (GraphPad Software, Boston, MA, USA).

## 3. Results

### 3.1. Chemical Synthesis of the Compounds

All synthesized compounds were obtained by the Hantzsch reaction as shown in Scheme 1. The identity of the compounds and their purity were confirmed by spectral analyses: MS, NMR, and IR. Their graphical depictions are presented in the Supplementary Materials.

### 3.2. Antiradical, Electron Transfer, and Ferrous Ion Chelation Assays

#### 3.2.1. Antiradical Assays

The results of the DPPH^•^ and ABTS^•+^ radical-scavenging assays by compounds **3a–j** are depicted in Table 2, expressed as their half-maximal inhibitory concentrations (IC_50_), along with the reference compounds, ascorbic acid and Trolox. 

#### 3.2.2. Electron Transfer Assays

The results of the evaluation of the electron transfer capacity of the compounds **3a–j**, performed using four methods (TAC, RP, FRAP, and CUPRAC), involving various oxidizing agents and environment scans be found in Table 3. The results are presented in the form of molar equivalents (Eq) of the control obtained by using Equation (2), mentioned previously.

#### 3.2.3. Ferrous Ion Chelation Assay

To evaluate the capacity of compounds **3a–j** to chelate ferrous ions, as a complementary antioxidant mechanism, a chelation assay using ferrozine as a chromogenic chelator was performed, using EDTA-NA_2_ as a reference. The result of the ferrous ion chelation assay is presented in Table 4.

### 3.3. In Silico Studies

#### 3.3.1. Theoretical Quantum Calculations 

For all compounds, we computed the HOMO and LUMO in vacuum, non-polar solvent, and water (Table 5). Additionally, for both phenol groups, we conducted the calculation of the Bond Dissociation Enthalpy (BDE) in all three environments (Table 6). Depictions of the HOMO and LUMO of compounds **3a–j** are illustrated in Table S1, and the electrostatic potential map for compounds **3a–j** is presented in Table S2, both being available in the Supplementary Materials. Full thermodynamic data computed for compounds **3a–j** are available in Tables S3–S5 in the Supplementary Materials.

We wanted to see how the substitution from position 2 of the thiazole influences the antioxidant activity of the catechol nucleus from position 4 of the thiazole, since we have a conjugation on the entire molecule, passing through the central thiazole. Therefore, the presence of a substituent or nucleus with some kind of electronic effect can influence the antioxidant activity of the catechol and/or the energy level in the molecule, which is a source of electrons when we have an oxidizing agent in the environment.

#### 3.3.2. Molecular Properties with Influence on the Pharmacokinetics of Compounds

The molecular properties of compounds **3a–j** were computed using in silico methods and are summarized in Table 7. The tested compounds have a molecular weight between 207.25 and 366.46, with all of them under 500, not violating the rule proposed by Lipinski [48]. Compounds **3i** and **3j** have the highest molecular weight, because of the presence of the supplementary thiazole ring in their structure, and compound **3f** has three methoxy groups. Regarding the number of rotatable bonds, according to the rule proposed by Lipinski, it should be less than 10, and according to the data obtained, all tested compounds adhere to this rule. The number of acceptor bonds varies depending on the nature of the radical located in position 2 of the thiazole, while the number of hydrogen donor bonds remains constant for all compounds, being determined by the two phenolic OH groups.

The TPSA value remains constant for compounds **3a–e** due to their similar structures and the presence of a single nitrogen and sulfur atom in each structure. Starting with compounds **3g** and **3h**, the TPSA value increases due to the introduction of the pyridyl moiety into the molecule, which adds a nitrogen atom in each case. Additionally, compound **3f** exhibits a high TPSA value owing to the presence of three methoxy groups in its structure. The highest values are recorded for compounds **3i** and **3j**, which feature dithiazole rings in their molecular structures.

The values of MLogP indicate balanced lipophilicity, and the compounds’ solubility in water varies from soluble to moderately soluble according to the data provided in Table 7.

### 3.4. Cytotoxicity Studies 

**Figure 1:**
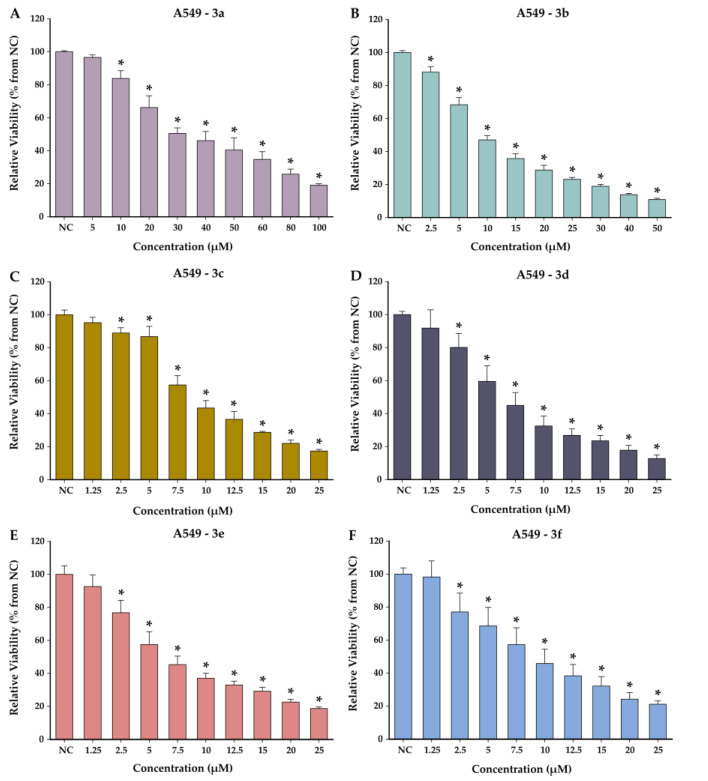
Cytotoxic effects of **3a** (**A**), **3b** (**B**), **3c** (**C**), **3d** (**D**), **3e** (**E**), and **3f** (**F**) after 48 h exposure in A549 cells. The results are represented as relative means ± SDs of three biological replicates. Data are expressed as relative values compared to the negative control (NC) (100%). Asterisks (*) indicate significant differences (*p* < 0.05) compared to NC.

**Figure 2:**
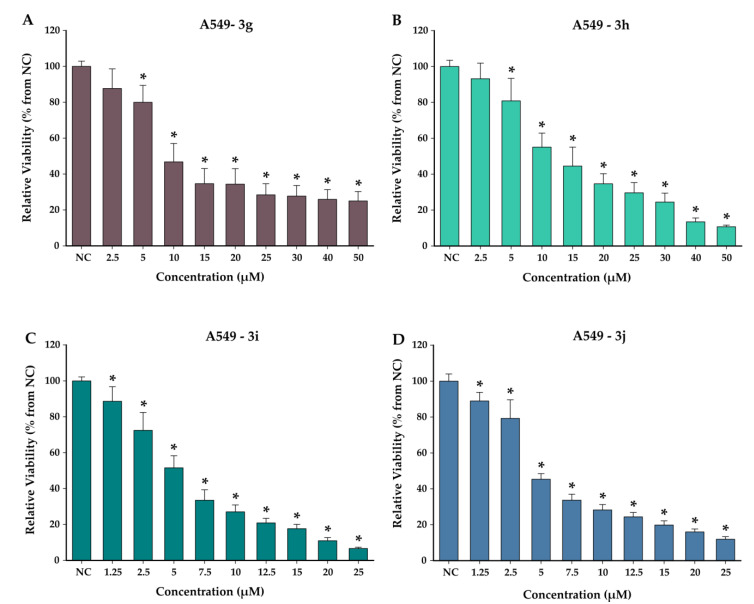
Cytotoxic effects of **3g** (**A**), **3h** (**B**), **3i** (**C**), and **3j** (**D**) after 48 h exposure in A549 cells. The results are represented as relative means ± SDs of three biological replicates. Data are expressed as relative values compared to the negative control (NC) (100%). Asterisks (*) indicate significant differences (*p* < 0.05) compared to NC.

**Figure 3:**
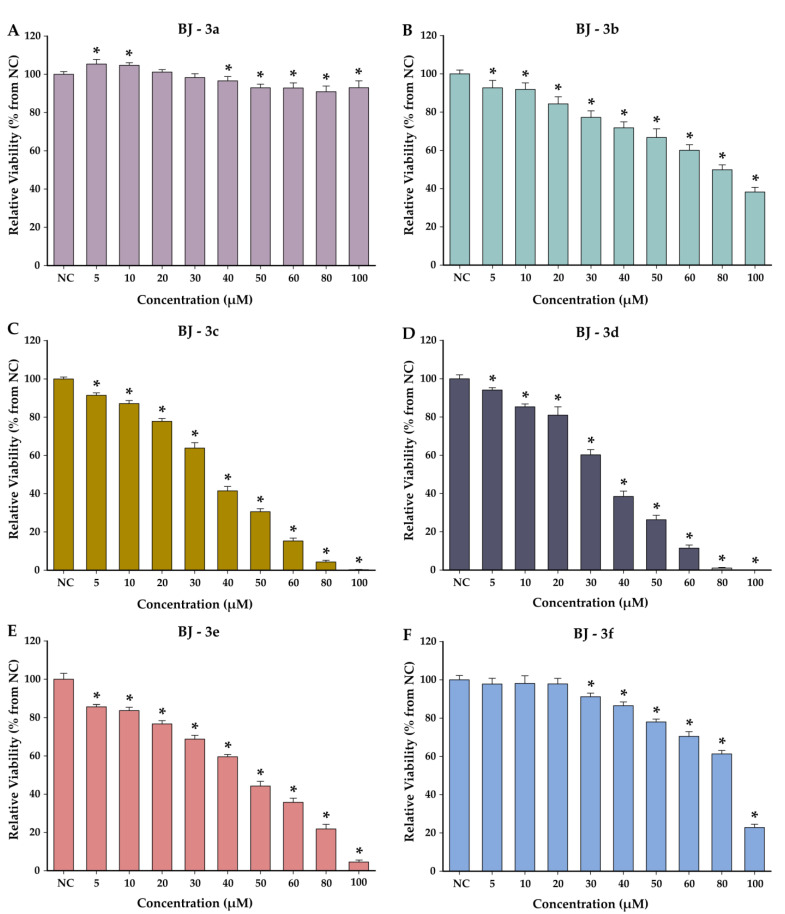
Cytotoxic effects of **3a** (**A**), **3b** (**B**), **3c** (**C**), **3d** (**D**), **3e** (**E**), and **3f** (**F**) after 48 h exposure in BJ cells. The results are represented as relative means ± SDs of three biological replicates. Data are expressed as relative values compared to the negative control (NC) (100%). Asterisks (*) indicate significant differences (*p* < 0.05) compared to NC.

**Figure 4:**
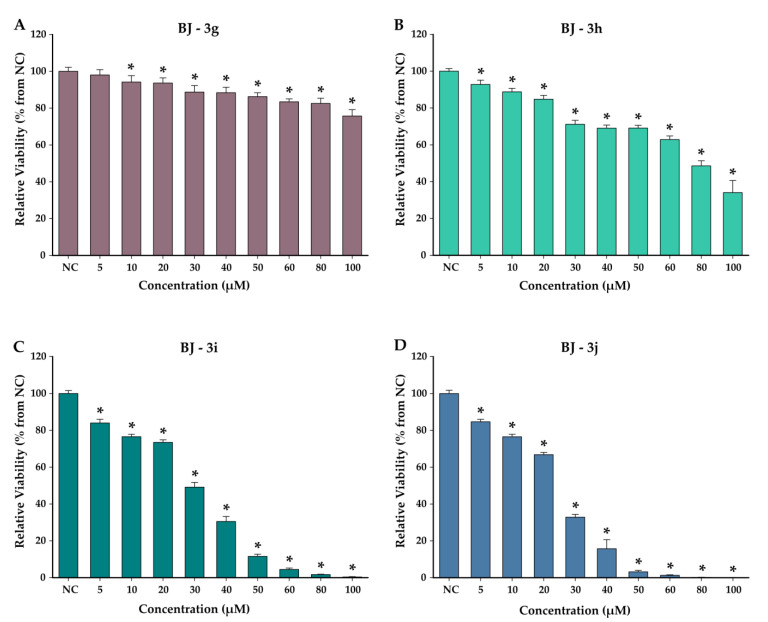
Cytotoxic effects of **3g** (**A**), **3h** (**B**), **3i** (**C**), and **3j** (**D**) after 48 h exposure in BJ cells. The results are represented as relative means ± SDs of three biological replicates. Data are expressed as relative values compared to the negative control (NC) (100%). Asterisks (*) indicate significant differences (*p* < 0.05) compared to NC.

The cytotoxicity of the synthesized compounds was evaluated on the human non-small lung cancer A549 cell line, in parallel with the normal BJ cell line. The effects were first evaluated in a concentration range from 1.25 to 100 µM for both cell lines after a 48 h exposure (Figure 1, Figure 2, Figure 3 and Figure 4). Depending on the obtained results, further testing concentrations were selected. As several synthesized compounds displayed increased cytotoxic effects on the malignant cell line, the concentrations were adjusted to allow for proper IC_50_ value calculation. Gefitinib, a chemotherapeutic drug with an indication in non-small lung cancer, was selected as a reference drug for the anticancer potential comparison. The choice of using gefitinib as reference anticancer agent was made according to ESMO (European Society for Medical Oncology) guidelines, which recommend gefitinib as the first-line treatment for non-small lung cancer in EGFR-mutated patients [52]. A supplementary strengthening reasoning for this choice is previous reports in the literature where 4-substituted thiazoles with structural similarity to our compounds were found to be potential anticancer agents via EGFR inhibition, the mechanism of gefitinib [53,54]. In the current assay, gefitinib was used for its anticancer properties, as it is known that it has no documented redox properties per se.

**Figure 5:**
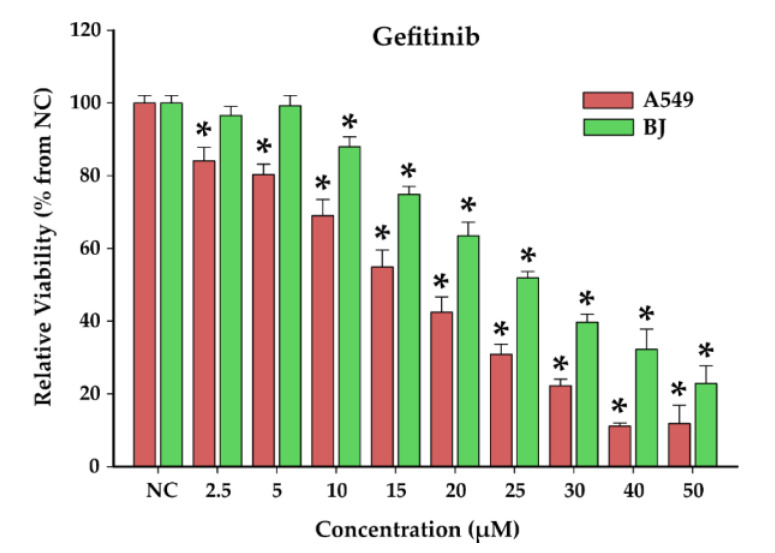
Cytotoxic effect of gefitinib after 48 h exposure in A549 and BJ cells. The results are represented as relative means ± SDs of three biological replicates. Data are expressed as relative values compared to the negative control (NC) (100%). Asterisks (*) indicate significant differences (*p* < 0.05) compared to NC.

Based on the AB data obtained, gefitinib displayed an IC_50_ value of 15.93 µM on A549 cells and 25.50 µM on BJ cells (Table 8, Figure 5). The selectivity index (SI) of gefitinib calculated as the ratio between the IC_50_ values obtained on cancerous and normal cells was 1.60 (Table 8). 

In comparison with gefitinib, apart from compound **3a** that had an IC_50_ value of 31.53 µM on A549 cells and no significant toxicity on BJ cells, all synthesized compounds displayed a higher cytotoxic potential. Additionally, the selectivity indexes of the synthesized compounds were higher than the one of gefitinib, indicating an increased selectivity and cytotoxicity toward the cancerous phenotype. On A549 cells, the potency of the compounds varied in the order **3j** > **3i** > **3d** > **3e** > **3g** > **3c** > **3f** > **3b** > **3h** > **3a**. Between all tested compounds, **3g** displayed one of the lowest IC_50_ values on A549 cells, while also displaying relatively low toxicity toward the normal cells. The calculated selectivity index for this compound was higher than 13.62 (Table 8), with the viability of BJ cells decreasing by approximately 20% at the highest tested dose.

## 4. Discussion

### 4.1. Chemical Synthesis of the Compounds

Starting from the structural model of compounds **3a**, **3b**, and **3c** (Scheme 1) already reported in the literature [22,23,24,25], we continued the development of such molecules. The synthesized compounds with a thiazolyl–catechol structure were obtained by the Hantzsch reaction according to the literature [12], starting from 4-chloroacetyl-catechol (**2**) and various thioamides (**1a–j**) as shown in Scheme 1. 

The structural identity of each compound was confirmed initially by the MS spectra, where the corresponding molecular peak was identified (Supplementary Figures S22–S28). According to the recorded ^1^H-NMR spectra (Supplementary Figures S8–S14), the structural identity of all the analyzed compounds was confirmed. The thiazolyl–catechol scaffold was similarly found in all the recorded spectra with signals in the aromatic region between 6 and 8 ppm. The H atom from position 5 of the thiazole appears as a singlet in all spectra around the value of 7.7 ppm. For the catechol nucleus, three signals are identified, one as a doublet at 6.8 ppm (*J* = 8 Hz), a second doublet at 7.4 ppm (*J* = 2 Hz), and the third signal being a doublet of doublets with an average value at 7.3 ppm (*J*_1_ = 2 Hz, *J*_2_ = 8 Hz). The fragment in position 2 of the thiazole, which is variable in our series of compounds, is also found in the aromatic region, apart from compounds **3f**, **3e**, and **3j**, which also have signals at values between 6 and 9 ppm and between 2 and 4 ppm for the aliphatic region.

Regarding the ^13^C-NMR spectra (Supplementary Figures S15–S21), the signals given by C atoms were found appropriately in the aromatic zone with values ranging from 100 to 170 ppm, and for compounds **3f**, **3e**, and **3j**, which contain aliphatic carbon atoms, the signals were found in the corresponding aliphatic zone, between 20 and 60 ppm.

The spectral data recorded for compounds **3a–j** matched the proposed structures. The recorded IR spectra for compounds **3a–j** displayed the expected signals, which are detailed in the Supplementary Materials, Figures S1–S7. For all synthesized compounds, the two signals from the phenolic OH groups can be observed between 3000 and 3600 cm^−1^. For compounds containing halogen atoms (**3c** and **3d**), the signals from these atoms can be seen at 613 cm^−1^ (C-Br) and 750 cm^−1^ (C-Cl). The methoxy groups in compound **3f** are found at 1080, 1021, 1252, and 1265 cm^−1^, signals specific for aryl–alkyl ethers. 

### 4.2. Antiradical, Electron Transfer, and Ferrous Ion Chelation Assays

In the current research, we employed more antioxidant assays: radical-scavenging techniques (DPPH and ABTS), electron transport techniques (TAC, RP, FRAP, and CUPRAC), and a supplementary assay (chelation of ferrous ions). All the performed assays are designed to measure the antioxidant capacity of tested compounds, but they have significant differences between them such as environmental factors of the assay, reaction mechanism, or strength of the oxidizing agent [29,55,56,57]. 

Regarding the differences between the antiradical assays, it is worth mentioning the environmental differences between the two assays—ABTS is performed in water, while DPPH is performed in ethanol. The solvent, due to its close interaction with the analyzed compounds, can influence their antioxidant behavior.

In the electron transfer assays, the TAC assay uses Mo^6+^ as an oxidizing agent at 95 °C for 1.5 h, RP uses Fe^3+^ as an oxidizing agent at 50 °C for 20 min in pH = 6.6, FRAP uses Fe^3+^ as an oxidizing agent at room temperature for 30 min but at pH = 3.6, and CUPRAC uses Cu^2+^ as an oxidizing agent in pH = 7.

The chelation of the ferrous ions is not an antioxidant assay per se, but due to the involvement of ferrous ions in the generation of radicals via Fenton reactions, a supplementary chelation potential for ferrous ions of an antioxidant compound would be beneficial for its activity.

In research involving antioxidants, the use of more assays allows for a comprehensive assessment of the antioxidant capacity of compounds under different conditions and mechanisms. Complementary techniques in antioxidant research ensure a thorough and reliable assessment of the antioxidant capacity of the compounds, cover different mechanisms of action, and provide validation and cross-verification of results.

This can be related to biological systems, where the interactions between antioxidants and oxidants are complex, and the use of more complementary assays can better simulate and clarify the behavior of antioxidants in different contexts. 

#### 4.2.1. Antiradical Assays 

In the DPPH test, as can be seen in Table 2, the most active compound was **3g** with the lowest IC_50_ value. This fact might be due to the presence of the 4-pyridyl fragment in position 2 of the thiazole, which confers a better capacity to neutralize free radicals. The second most active compound was **3h**, the positional isomer of compound **3g**, which also has a 2-pyridyl fragment in its structure that contributes to the antiradical activity. The difference in antiradical activity between the other compounds is not significant, given the relatively close IC_50_ values. This indicates that the catechol moiety exhibits antioxidant activity regardless of the fragment present in position 2 of the thiazole.

On the other hand, in the ABTS test, compound **3g** stood out with a significantly lower IC_50_ compared to the reference substance, which supports the ability of the 4-pyridyl fragment to contribute to the antioxidant activity. Unlike **3g**, compound **3h**, which is its positional isomer, having the nitrogen atom from pyridine in position 2, shows a weaker antiradical activity than **3g** due to the different electron distribution, which can influence the antioxidant activity. According to the IC_50_ values presented in Table 2, the antiradical activity of the other tested compounds decreased in the following order: **3a** > **3d** > **3f** > **3h** > **3e** > **3b** > **3i** > **3c** > **3j**. Compound **3j** was the least active of the tested compounds, having an IC_50_ higher than that of Trolox, most likely due to the voluminous radical located in position 2 of the thiazole ring, which can prevent the neutralization of free radicals. 

#### 4.2.2. Electron Transfer Assays

In the TAC test, we measured the reduction of Mo^+6^ to Mo^+5^ by recording the intensity of the blue-green color of the product formed after the transfer of electrons. According to the results presented in Table 3, compound **3h** had the highest electron transfer capacity among the tested compounds, due to the 2-pyridyl fragment from its structure. Unlike its isomer, compound **3g** showed moderate electron transfer activity due to the nitrogen atom located in position 4, which determines a different electron distribution on the molecular surface. Also, compounds **3f** and **3b** presented a good electron transfer capacity, with the others having moderate activity. Among all the compounds tested, **3a** and **3j** had the weakest electron transfer capacity compared to the ascorbic acid used as a reference.

The reducing power of the compounds **3a–j** was tested by their ability to reduce ferric ions from potassium ferricyanide, forming ferrocyanide, which gives a blue complex. Their reducing capacity was compared with two standards, ascorbic acid and Trolox. According to the data shown in Table 3, all tested compounds had a superior reducing power to the reference substances. Compound **3h** exhibited the strongest reducing capacity, followed by **3g**, due to the presence of the 2-pyridyl and 4-pyridyl fragments in their structure. For the other compounds, the reducing power decreases in the order **3j** > **3f** > **3a** > **3e** > **3i** = **3d** > **3b** > **3c**. Compound **3c** has the weakest reducing power of the entire series, but it is not weaker than those of the reference antioxidants.

The results of the FRAP test presented in Table 3 show that all compounds, except **3a**, had a better electron transfer capacity than Trolox, used as a reference. Compound **3g** also stands out in this test with a superior activity compared to the other tested compounds, having the most prominent activity. The electron transfer capacity within this test decreases in the order **3i** > **3c** > **3d** > **3e** = **3b** > **3j** = **3f** > **3h** > **3a**. The difference between **3g** and **3h** is most likely due to the position of the pyridyl nitrogen atom.

Regarding the CUPRAC test, where Trolox was used as a reference substance, all the tested compounds had good electron transfer capacity. Compound **3g** shows the highest electron transfer capacity among all tested compounds, due to the substituent in position 2 of the thiazole. Compound **3j** exhibited the weakest electron transfer capacity in this test, because of the voluminous fragment in position 2 of the thiazole, which inhibits proton transfer from catechol. The order in which the antioxidant activity of the other compounds decreases is **3a** > **3e** > **3f** > **3d** > **3c** > **3b** > **3h** > **3i**.

#### 4.2.3. Ferrous Ion Chelation Assay

Chelation of ferrous ions can be useful in combating oxidative stress because iron ions (Fe^2+^) play a significant role in the apparition of ROS by a Fenton reaction. Chelation of the free ferrous ions can help restore redox balance in cells and tissues, thereby reducing oxidative stress and its associated detrimental effects on health. The evaluated compounds **3a–j** exhibited no chelation activity within the tested concentration range for the ferrous ions when compared to EDTA-Na_2_, which is well established as an effective chelator for Fe^+2^ ions. 

### 4.3. In Silico Studies

#### 4.3.1. Theoretical Quantum Calculations 

Computational methods are currently employed to theoretically explain and predict the antioxidant potential of compounds in correlation with their molecular behavior. To establish connections and to explain the results of the performed antioxidant assays in relation to their structure and substitution of these compounds, we have performed an investigation focused on the influence of the structures on their activity. 

In compound **3a**, substituted with just a methyl residue in position 2 of the thiazole ring, the HOMO and LUMO are overlapping. In all other compounds, the HOMO is found over the 4-thiazolyl-catechol region, while the LUMO was found over the 2-thiazolyl-(het)arene region. The highest energy levels were identified for the HOMO in a vacuum and non-polar environment for compounds **3c**, **3d**, and **3g**, with an electronegative moiety on the substituent—**3c** (*p*-Cl), **3d** (*p*-Br), and **3g** (4Py). In water, due to the interaction with the solvent, HOMO levels from the current series were the highest for compounds **3g**, **3h**, **3e**, and **3i**, compounds having substitution with a heteroarene as a common feature (**3g**, **3h**, and **3i**) or a *meta*-tolyl. The analysis of variation in the energy of the HOMO according to the solvent indicates that the compounds with electron-donating groups have a moderate (compounds **3a**, **3b**, **3f**, and **3j**—0.10–0.14 eV) or a higher (compounds **3h**, **3e** and **3i**—0.24–0.26 eV) increase in the HOMO levels. The variations in the LUMO levels are similar to the HOMO ones. The HOMO-LUMO gap slightly changes due to the presence of water when compared to vacuum, with significant changes for compound **3g** (0.06 eV increase) and compound **3h** (0.09 eV decrease), while for most compounds, the changes are negligible. The smallest HOMO-LUMO gap was identified for compound **3g** from the current series of compounds in all environments tested, which means the respective compound can readily donate an electron, being favorable for its antioxidant activity. A smaller gap usually suggests higher chemical reactivity and lower kinetic stability, while a larger gap indicates the opposite. For a good antioxidant, a smaller HOMO-LUMO gap is desired [58,59]. 

This leads to a similar finding for hardness (η) and chemical potential (µ), derived from the HOMO and LUMO. In all environments, from the current series for compound **3g**, the lowest hardness was found (between 1.80 eV in water and 1.83 eV in vacuum) as well as the lowest chemical potential (−3.70 eV in all solvents). Interestingly, the effect of water on the hardness and chemical potential is significant for compounds **3h**, **3i**, and **3j**, when compared to the vacuum or non-polar environment.

The HOMO-LUMO gap, hardness, and chemical potentials remain relatively consistent across different environments for some compounds, but some effects due to the solvation are worth noting. This suggests that the solvent environment can alter the electronic properties of some of the compounds and their antioxidant behavior. When taking into account the heterolytic breaking of the OH bonds, it can be seen that the OH in *para* would release a hydrogen atom more easily than the OH in *meta*, independent of the molecule and environment, with the minimum difference in enthalpy for the two sites being at least 1.95 kcal/mol (compound **3h** in vacuum), reaching a maximum difference of 2.75 kcal/mol (compound **3a** in water). 

Overall, in a non-polar environment, the radicalization of the OH in *para* would take place with approximately 0.5 kcal/mol on average more energy than in a vacuum, but a higher increase in the enthalpy is found in water (approximately 3.5 kcal/mol in average), perfectly understandable due to the properties of the water.

Analysis of the BDEs of the OH groups revealed low values, indicating they are easy to break in a homolytic way, releasing hydrogen atoms. This finding, consistent with previously reported data from our group and others, suggested that the synthesized compounds possessed good antiradical activity [17,44]. Between a specific OH group of the compounds in a specific environment, low differences in terms of BDE were identified, suggesting that the substitution in position 2 of the thiazole has a moderate to low influence on the activity of the antiradical activity of the phenol OH groups from the catechol. 

#### 4.3.2. Molecular Properties with Influence on the Pharmacokinetics of Compounds

We conducted a SwissADME assessment to evaluate the physicochemical properties of the compounds and ascertain whether they adhere to Lipinski’s rule of 5.

All compounds presented 0 violations of the Lipinski rules, which suggests that compounds meeting these criteria have a higher probability of exhibiting desirable pharmacokinetic properties, such as good oral bioavailability and permeability. Therefore, Lipinski’s rule of 5 is widely used in early-stage drug discovery as a guideline to prioritize and optimize lead compounds. 

### 4.4. Cytotoxicity Studies on Normal and Cancerous Cell Lines

Another evaluation of compounds **3a–j** was performed to assess the cytotoxicity on normal fibroblast cells (BJ) and a lung cancer cell line (A549). After exposing the cells to compounds **3a–j** for 48 h, the cytotoxic activity was measured, showing that the compounds were more toxic to A549 cells than to BJ cells (Table 8, Figure 1, Figure 2, Figure 3 and Figure 4). 

Regarding the BJ cells, higher IC_50_ values indicate less toxicity. According to the IC_50_ values in Table 8, compounds **3a** and **3g** were the least toxic to BJ cells, while compound **3j** was the most toxic. The selectivity index (SI) was calculated for all compounds to evaluate their specificity for cancer cells. A higher SI indicates a greater specificity for the target. All compounds except **3a** and **3c** had an SI greater than 5, suggesting a higher likelihood of cytotoxic effects on cancer cells. Compound **3g** had the highest SI (>13.62), indicating a strong preference for the A549 cell line and a potential therapeutic utility.

Compound **3j** showed the highest potency on A549 cells (IC_50_ = 4.28 μM), likely due to its *p*-tolyl group enhancing its lipophilicity and cellular permeability. The dithiazole moiety also contributes to the stability and effective cellular interactions. Compound **3i**, which has a benzyl group, also showed strong activity, although not as potent as **3j**. On the other hand, compound **3a** had the highest IC_50_ value (31.53 μM), indicating lower potency due to its single thiazole ring and limited conjugation.

The differences in IC_50_ values between compounds **3g** and **3h** on A549 cells can be attributed to the positional effects of the pyridine substituent. Compound **3g**, with the 4-pyridil fragment, showed better electron delocalization and lower steric hindrance, resulting in a lower IC_50_ value. Compound **3d** outperformed **3c**, probably, due to the stronger van der Waals interactions and favorable electronic effects of the bromine atom.

Compared to gefitinib, used as a reference drug, all compounds except from **3a** exhibited lower IC_50_ values on the A549 cell line. Additionally, the synthesized compounds demonstrated lower toxicity on BJ cells and higher selectivity indexes compared to gefitinib. Notably, compound **3j** has an IC_50_ that is 3.7 times lower than that of gefitinib, indicating significant cytotoxic activity on A549 cells compared to the reference drug. Compounds **3j** and **3i** exhibited the highest potency against A549 cells, while compound **3g** demonstrated exceptional selectivity compared to the reference drug. The substituents seem to have a significant influence on the biological activity, as we may see that the presence of dithiazole, pyridyl, and bromine moieties enhancing both potency and selectivity.

## 5. Conclusions 

Following this study, a series of thiazolyl–catechol compounds were synthesized and subjected to comprehensive in silico analyses and in vitro assays to evaluate their antioxidant and cytotoxic properties. The in silico analyses indicated that the antioxidant efficacy of these compounds is not influenced by their substituents, which do not significantly affect the electron distribution across the molecule and the stability of the O-H bonds from the phenol groups of catechol. Some antioxidant effects were brought in the compounds derived from pyridine, which can be attributed to the respective ring itself, due to its interesting electron properties. 

The in vitro tests demonstrated that compound **3g** exhibited superior antioxidant activity, with significantly lower IC_50_ values compared to the reference antioxidants, indicating high efficiency in radical scavenging and electron transfer. Compound **3h** also displayed notable antioxidant activity, but to a lesser extent. The cytotoxicity assays on BJ human fibroblast and A549 lung cancer cell lines revealed that compound **3g** had the lowest toxicity on normal cells and the highest cytotoxic effect on cancer cells, with a selectivity index of 13.62, indicating a high selectivity for cancer cells. Several compounds outperformed the reference drug, gefitinib, indicating promising candidates for further optimization and development as potential anticancer agents. 

These promising results suggest that thiazolyl–catechol compounds hold significant potential for further development in the field of medicinal chemistry. Future research could explore modifications in position 2 of the thiazole ring to enhance other physicochemical and biological properties. 

## Figures and Tables

**Table 1:** The R fragment from the position 2 of the thiazole ring in compounds **3a–j**.

Compound	R	Compound	R
**1a, 3a**	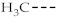	**1f, 3f**	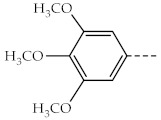
**1b, 3b**	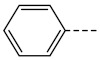	**1g, 3g**	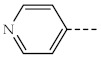
**1c, 3c**	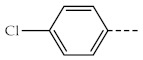	**1h, 3h**	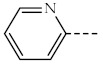
**1d, 3d**	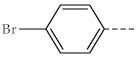	**1i, 3i**	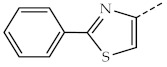
**1e, 3e**	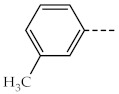	**1j, 3j**	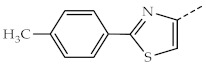

**Table 2:** The half-maximal inhibitory concentrations (IC_50_) registered for compounds **3a–j**, against the DPPH^•^ and ABTS^•+^ (µM).

Compound	DPPH^•^	ABTS^•+^
**3a**	51.31	12.01
**3b**	50.19	14.03
**3c**	47.83	15.74
**3d**	48.34	12.62
**3e**	46.76	13.88
**3f**	54.61	13.36
**3g**	36.69	7.59
**3h**	40.30	13.49
**3i**	45.86	14.08
**3j**	49.59	17.66
**Ascorbic acid**	53.49	NT
**Trolox**	38.10	15.87

NT = not tested.

**Table 3:** Results of the electron transfer assays performed for compounds **3a–j**, expressed as Eq activity of ascorbic acid and/or Trolox.

Compound	TAC	RP		FRAP	CUPRAC
	Eq Ascorbic Acid	Eq Ascorbic Acid	Eq Trolox	Eq Trolox	Eq Trolox
**3a**	0.38	1.73	1.44	0.95	3.17
**3b**	1.00	1.60	1.33	1.07	2.27
**3c**	0.83	1.47	1.22	1.10	2.38
**3d**	0.71	1.63	1.35	1.09	2.62
**3e**	0.91	1.64	1.37	1.07	3.13
**3f**	1.22	1.75	1.46	1.06	3.05
**3g**	0.64	2.04	1.69	1.63	3.60
**3h**	1.34	2.06	1.71	1.03	2.13
**3i**	0.51	1.63	1.35	1.14	1.84
**3j**	0.36	1.81	1.50	1.06	1.61

**Table 4:** The ferrous ion chelation percent, depending on the concentration of the tested compounds (µM).

Compound	2 µM	1.5 µM	1 µM	0.5 µM	0.25 µM
**3a**	-	-	-	-	-
**3b**	-	-	-	-	-
**3c**	-	-	-	-	-
**3d**	-	-	-	-	-
**3e**	-	-	-	-	-
**3f**	-	-	-	-	-
**3g**	-	-	-	-	-
**3h**	-	-	-	-	-
**3i**	-	-	-	-	-
**3j**	-	-	-	-	-
**EDTA–Na_2_**	96.20	86.52	73.41	55.24	47.21

- represents negligible activity.

**Table 5:** The energy level of the HOMO, LUMO, and the derived descriptors HOMO-LUMO gap, hardness (η), and chemical potential (µ) in vacuum, non-polar environment, and water for compounds **3a–j** (eV).

	Vacuum					Non-Polar					Water				
	HOMO	LUMO	Gap	η	µ	HOMO	LUMO	Gap	η	µ	HOMO	LUMO	Gap	η	µ
**3a**	−5.31	−0.84	4.47	2.24	−3.08	−5.40	−0.88	4.52	2.26	−3.14	−5.42	−0.89	4.53	2.27	−3.16
**3b**	−5.32	−1.42	3.90	1.95	−3.37	−5.40	−1.50	3.90	1.95	−3.45	−5.42	−1.51	3.91	1.96	−3.47
**3c**	−5.43	−1.65	3.78	1.89	−3.54	−5.44	−1.65	3.79	1.90	−3.55	−5.44	−1.65	3.79	1.90	−3.55
**3d**	−5.44	−1.68	3.76	1.88	−3.56	−5.44	−1.68	3.76	1.88	−3.56	−5.45	−1.68	3.77	1.89	−3.57
**3e**	−5.29	−1.39	3.90	1.86	−3.44	−5.37	−1.47	3.90	1.95	−3.42	−5.54	−1.60	3.94	1.97	−3.57
**3f**	−5.27	−1.35	3.92	1.96	−3.31	−5.37	−1.49	3.88	1.94	−3.43	−5.39	−1.51	3.88	1.94	−3.45
**3g**	−5.53	−1.87	3.66	1.83	−3.70	−5.50	−1.89	3.61	1.81	−3.70	−5.50	−1.90	3.60	1.80	−3.70
**3h**	−5.31	−1.61	3.70	1.85	−3.46	−5.39	−1.68	3.71	1.86	−3.54	−5.57	−1.78	3.79	1.90	−3.68
**3i**	−5.32	−1.64	3.68	1.84	−3.48	−5.41	−1.70	3.71	1.86	−3.56	−5.56	−1.83	3.73	1.87	−3.70
**3j**	−5.29	−1.58	3.71	1.95	−3.34	−5.40	−1.67	3.73	1.87	−3.54	−5.43	−1.69	3.74	1.87	−3.56

**Table 6:** The BDE for the OH phenol groups from compounds **3a–j** in a vacuum, non-polar environment, and water (kcal/mol).

Compound	Vacuum		Non-Polar		Water	
	*para*	*meta*	*para*	*meta*	*para*	*meta*
**3a**	66.51	68.76	67.37	70.01	69.87	72.62
**3b**	66.72	68.94	67.75	70.25	70.26	72.83
**3c**	67.05	69.09	67.85	70.28	70.34	72.82
**3d**	67.05	69.12	67.96	70.37	70.47	72.97
**3e**	66.63	68.82	66.07	68.32	70.17	72.75
**3f**	66.69	68.93	67.71	70.19	70.25	72.81
**3g**	67.34	69.33	68.30	70.54	70.81	73.12
**3h**	66.72	68.67	66.18	68.24	70.26	72.71
**3i**	66.90	68.96	66.33	68.57	70.42	72.92
**3j**	66.83	68.90	67.86	70.22	70.39	72.83

**Table 7:** The molecular properties of the compounds **3a–j** with strong influences on their pharmacokinetics.

Compound	MW	Rotatable Bonds	HBA	HBD	TPSA (Å^2^)	MLogP	Water Solubility (µg/mL)	Lipinski Violations
**3a**	207.25	1	3	2	81.59	0.61	1.69	0
**3b**	269.32	2	3	2	81.59	2.01	1.38	0
**3c**	303.76	2	3	2	81.59	2.52	4.19	0
**3d**	348.21	2	3	2	81.59	2.65	2.30	0
**3e**	283.34	2	3	2	81.59	2.25	7.63	0
**3f**	359.40	5	6	2	109.28	1.05	1.32	0
**3g**	270.31	2	4	2	94.48	0.90	6.47	0
**3h**	270.31	2	4	2	94.48	0.90	6.19	0
**3i**	352.43	3	4	2	122.72	1.97	2.18	0
**3j**	366.46	3	4	2	122.72	2.20	1.16	0

MW = “molecular weight”; HBA = “hydrogen bond acceptors”; HBD = “hydrogen bond donors”.

**Table 8:** The IC_50_ values (µM) (± SD) after exposure in A549 and BJ cells to compounds **3a–j** for 48 h. The selectivity index was calculated as the ratio between the calculated IC_50_.

Compound	IC_50_ (µM) (±SD)		Selectivity Index
	A549	BJ	
**3a**	31.53 (±6.71)	>100	>3.17
**3b**	8.91 (±0.62)	78.61 (±9.52)	8.82
**3c**	8.16 (±0.46)	35.83 (±2.92)	4.39
**3d**	6.08 (±1.22)	34.44 (±2.50)	5.66
**3e**	6.46 (±1.16)	46.77 (±5.52)	7.23
**3f**	8.76 (±1.48)	82.61 (±3.68)	9.43
**3g**	7.34 (±1.02)	>100	>13.62
**3h**	13.13 (±1.16)	76.72 (±9.92)	5.84
**3i**	5.18 (±0.70)	29.39 (±2.08)	5.67
**3j**	4.28 (±0.37)	24.25 (±1.60)	5.67
**Gefitinib**	15.93 (±0.50)	25.50 (±0.51)	1.60

## Data Availability

Data are contained within the article or Supplementary Materials.

## Supplementary Materials

The following supporting information can be downloaded at https://www.mdpi.com/article/10.3390/antiox13080937/s1. Scheme S1: The synthesis route for obtention of intermediate thioamides **1f**, **1i**, and **1j**, reported in our group’s previous reports. Figures S1–S7: The IR spectra for compounds 3d–j. Figures S8–S14: The 1H-NMR spectrum for compounds 3d–j. Figures S15–S21: The 13C-NMR spectrum for compounds 3d–j. Figures S22–S28: The MS spectrum for compounds 3d–j. Table S1: The depiction of HOMO and LUMO for the compounds 3a–j. Table S2: The electrostatic potential map for compounds 3a–j. Table S3: Thermodynamic data computed for compounds 3a–j in vacuum (T = 298.15 K, P = 1 atm). Table S4: Thermodynamic data computed for compounds 3a–j in nonpolar solvent (T = 298.15 K, P = 1 atm). Table S5: Thermodynamic data computed for compounds 3a–j in water (T = 298.15 K, P = 1 atm)

## References

[1] Sharifi-Rad M., Anil Kumar N.V., Zucca P., Varoni E.M., Dini L., Panzarini E., Rajkovic J., Tsouh Fokou P.V., Azzini E., Peluso I. (2020). Lifestyle, Oxidative Stress, and Antioxidants: Back and Forth in the Pathophysiology of Chronic Diseases. Front. Physiol..

[2] Valko M., Leibfritz D., Moncol J., Cronin M.T.D., Mazur M., Telser J. (2007). Free radicals and antioxidants in normal physiological functions and human disease. Int. J. Biochem. Cell Biol..

[3] Reuter S., Gupta S.C., Chaturvedi M.M., Aggarwal B.B. (2010). Oxidative stress, inflammation, and cancer: How are they linked?. Free Radic. Biol. Med..

[4] Trachootham D., Alexandre J., Huang P. (2009). Targeting cancer cells by ROS-mediated mechanisms: A radical therapeutic approach?. Nat. Rev. Drug Discov..

[5] Sosa V., Moliné T., Somoza R., Paciucci R., Kondoh H., LLeonart M.E. (2013). Oxidative stress and cancer: An overview. Ageing Res. Rev..

[6] Valko M., Rhodes C.J., Moncol J., Izakovic M., Mazur M. (2006). Free radicals, metals and antioxidants in oxidative stress-induced cancer. Chem. Biol. Interact..

[7] Klaunig J.E., Wang Z., Pu X., Zhou S. (2011). Oxidative stress and oxidative damage in chemical carcinogenesis. Toxicol. Appl. Pharmacol..

[8] Grivennikov S.I., Greten F.R., Karin M. (2010). Immunity, Inflammation, and Cancer. Cell.

[9] Mittal M., Siddiqui M.R., Tran K., Reddy S.P., Malik A.B. (2014). Reactive oxygen species in inflammation and tissue injury. Antioxid. Redox Signal..

[10] Biswas S.K. (2016). Does the Interdependence between Oxidative Stress and Inflammation Explain the Antioxidant Paradox?. Oxidative Med. Cell. Longev..

[11] Arshad M.F., Alam A., Alshammari A.A., Alhazza M.B., Alzimam I.M., Alam M.A., Mustafa G., Ansari M.S., Alotaibi A.M., Alotaibi A.A. (2022). Thiazole: A Versatile Standalone Moiety Contributing to the Development of Various Drugs and Biologically Active Agents. Molecules.

[12] Petrou A., Fesatidou M., Geronikaki A. (2021). Thiazole Ring—A Biologically Active Scaffold. Molecules.

[13] Singh A., Malhotra D., Singh K., Chadha R., Bedi P.M.S. (2022). Thiazole derivatives in medicinal chemistry: Recent advancements in synthetic strategies, structure activity relationship and pharmacological outcomes. J. Mol. Struct..

[14] Hussain T., Tan B., Yin Y., Blachier F., Tossou M.C.B., Rahu N. (2016). Oxidative Stress and Inflammation: What Polyphenols Can Do for Us?. Oxidative Med. Cell. Longev..

[15] Sajadimajd S., Bahramsoltani R., Iranpanah A., Kumar Patra J., Das G., Gouda S., Rahimi R., Rezaeiamiri E., Cao H., Giampieri F. (2020). Advances on Natural Polyphenols as Anticancer Agents for Skin Cancer. Pharmacol. Res..

[16] Gorlach S., Fichna J., Lewandowska U. (2015). Polyphenols as mitochondria-targeted anticancer drugs. Cancer Lett..

[17] Marc G., Stana A., Franchini A.H., Vodnar D.C., Barta G., Tertiş M., Şanta I., Cristea C., Pîrnău A., Ciorîţă A. (2021). Phenolic Thiazoles with Antioxidant and Antiradical Activity. Synthesis, In Vitro Evaluation, Toxicity, Electrochemical Behavior, Quantum Studies and Antimicrobial Screening. Antioxidants.

[18] Araniciu C., Palage M., Oniga S., Pirnau A., Verité P., Oniga O. (2013). Synthesis and characterization of some novel 5,2-and 4,2-bisthiazoles derivatives. Rev. Chim..

[19] Araniciu C., Pârvu A.E., Tiperciuc B., Palage M., Oniga S., Verité P., Oniga O. (2013). Synthesis and evaluation of the anti-inflammatory activity of some 2-(Trimethoxyphenyl)-4-R1-5-R2-Thiazoles. Dig. J. Nanomater. Biostruct..

[20] Araniciu C., Pârvu A.E., Palage M.D., Oniga S.D., Benedec D., Oniga I., Oniga O. (2014). The effect of some 4,2 and 5,2 bisthiazole derivatives on nitro-oxidative stress and phagocytosis in acute experimental inflammation. Molecules.

[21] Ali S.H., Sayed A.R. (2021). Review of the synthesis and biological activity of thiazoles. Synth. Commun..

[22] Narayana B., Ashalatha B.V., Vijaya Raj K.K., Suchetha Kumari N. (2006). Synthesis of some new 4-{2-[(aryl)amino]-1,3-thiazol-4-yl}benzene-1,2-diols as possible antibacterial and antifungal agents. Phosphorus Sulfur Silicon Relat. Elem..

[23] Wang W.L., Chai S.C., Huang M., He H.Z., Hurley T.D., Ye Q.Z. (2008). Discovery of inhibitors of Escherichia coli methionine aminopeptidase with the Fe(II)-form selectivity and antibacterial activity. J. Med. Chem..

[24] Alam M.A. (2023). Thiazole Derivates and Methods of Using the Same. WO Patent.

[25] Chihiro M., Komatsu H., Michiaki T., Yoichi Y. (2000). Superoxide Radical Inhibitors. U.S. Patent.

[26] Brand-Williams W., Cuvelier M.E., Berset C. (1995). Use of a free radical method to evaluate antioxidant activity. LWT Food Sci. Technol..

[27] Bedlovicová Z., Strapác I., Baláž M., Salayová A. (2020). A Brief Overview on Antioxidant Activity. Molecules.

[28] Theodosis-Nobelos P., Papagiouvannis G., Rekka E.A. (2023). Ferulic, Sinapic, 3,4-Dimethoxycinnamic Acid and Indomethacin Derivatives with Antioxidant, Anti-Inflammatory and Hypolipidemic Functionality. Antioxidants.

[29] Alam M.N., Bristi N.J., Rafiquzzaman M. (2013). Review on in vivo and in vitro methods evaluation of antioxidant activity. Saudi Pharm. J..

[30] Re R., Pellegrini N., Proteggente A., Pannala A., Yang M., Rice-Evans C. (1999). Antioxidant activity applying an improved ABTS radical cation decolorization assay. Free Radic. Biol. Med..

[31] Baig H., Ahmed D., Zara S., Aujla M.I., Asghar M.N. (2011). In Vitro Evaluation of Antioxidant Properties of Different Solvent Extracts of Rumex acetosella Leaves. Orient. J. Chem..

[32] Ramadan S.K., Elrazaz E.Z., Abouzid K.A.M., El-Naggar A.M. (2020). Design, synthesis and in silico studies of new quinazolinone derivatives as antitumor PARP-1 inhibitors. RSC Adv..

[33] Benzie I.F.F., Strain J.J. (1999). Ferric reducing/antioxidant power assay: Direct measure of total antioxidant activity of biological fluids and modified version for simultaneous measurement of total antioxidant power and ascorbic acid concentration. Methods in Enzymology.

[34] Özyürek M., Güçlü K., Apak R. (2011). The main and modified CUPRAC methods of antioxidant measurement. TrAC Trends Anal. Chem..

[35] Apak R., Güçlü K., Demirata B., Özyürek M., Çelik S., Bektaşoğlu B., Berker K., Özyurt D. (2007). Comparative Evaluation of Various Total Antioxidant Capacity Assays Applied to Phenolic Compounds with the CUPRAC Assay. Molecules.

[36] Dinis T.C.P., Madeira V.M.C., Almeida L.M. (1994). Action of phenolic derivatives (acetaminophen, salicylate, and 5-aminosalicylate) as inhibitors of membrane lipid peroxidation and as peroxyl radical scavengers. Arch. Biochem. Biophys..

[37] Sudan R., Bhagat M., Gupta S., Singh J., Koul A. (2014). Iron (FeII) Chelation, Ferric Reducing Antioxidant Power, and Immune Modulating Potential of Arisaema jacquemontii (Himalayan Cobra Lily). BioMed Res. Int..

[38] Turan B., Şendil K., Şengül E., Gültekin M.S., Taslimi P., Gulçin İ., Supuran C.T. (2016). The synthesis of some β-lactams and investigation of their metal-chelating activity, carbonic anhydrase and acetylcholinesterase inhibition profiles. J. Enzyme Inhib. Med. Chem..

[39] Manojlovic N.T., Vasiljevic P.J., Maskovic P.Z., Juskovic M., Bogdanovic-Dusanovic G. (2012). Chemical Composition, Antioxidant, and Antimicrobial Activities of Lichen Umbilicaria cylindrica (L.) Delise (Umbilicariaceae). Evid. Based Complement. Altern. Med..

[40] Hossen J., Pal T.K., Hasan T. (2022). Theoretical investigations on the antioxidant potential of 2,4,5-trihydroxybutyrophenone in different solvents: A DFT approach. Results Chem..

[41] Rodríguez S.A., Baumgartner M.T. (2014). Theoretical study of the reaction mechanism of a series of 4-hydroxycoumarins against the DPPH radical. Chem. Phys. Lett..

[42] Xue Y., Zheng Y., An L., Dou Y., Liu Y. (2014). Density functional theory study of the structure–antioxidant activity of polyphenolic deoxybenzoins. Food Chem..

[43] Giacomelli C., Miranda F.d.S., Gonçalves N.S., Spinelli A. (2004). Antioxidant activity of phenolic and related compounds: A density functional theory study on the O–H bond dissociation enthalpy. Redox Rep..

[44] Antonijević M.R., Simijonović D.M., Avdović E.H., Ćirić A., Petrović Z.D., Marković J.D., Stepanić V., Marković Z.S. (2021). Green One-Pot Synthesis of Coumarin-Hydroxybenzohydrazide Hybrids and Their Antioxidant Potency. Antioxidants.

[45] Molaei S., Tehrani A.D., Shamlouei H. (2023). Antioxidant activates of new carbohydrate based gallate derivatives: A DFT study. J. Mol. Liq..

[46] Daina A., Michielin O., Zoete V. (2017). SwissADME: A free web tool to evaluate pharmacokinetics, drug-likeness and medicinal chemistry friendliness of small molecules. Sci. Rep..

[47] Ertl P., Rohde B., Selzer P. (2000). Fast calculation of molecular polar surface area as a sum of fragment-based contributions and its application to the prediction of drug transport properties. J. Med. Chem..

[48] Lipinski C.A., Lombardo F., Dominy B.W., Feeney P.J. (2012). Experimental and computational approaches to estimate solubility and permeability in drug discovery and development settings. Adv. Drug Deliv. Rev..

[49] Delaney J.S. (2004). ESOL: Estimating Aqueous Solubility Directly from Molecular Structure. J. Chem. Inf. Comput. Sci..

[50] Topală T.L., Fizeşan I., Petru A., Castiñeiras A., Bodoki A.E., Oprean L.S., Escolano M., Alzuet-Piña G. (2024). Evaluation of DNA and BSA-Binding, Nuclease Activity, and Anticancer Properties of New Cu(II) and Ni(II) Complexes with Quinoline-Derived Sulfonamides. Inorganics.

[51] Șandor A., Fizeșan I., Ionuț I., Marc G., Moldovan C., Oniga I., Pîrnău A., Vlase L., Petru A.-E., Macasoi I. (2024). Discovery of A Novel Series of Quinazoline–Thiazole Hybrids as Potential Antiproliferative and Anti-Angiogenic Agents. Biomolecules.

[52] ESMO Lung & Chest Tumours. https://interactiveguidelines.esmo.org/esmo-web-app/toc/index.php?subjectAreaID=1&loadPdf=1.

[53] El-Naggar A.M., Zidan A., Elkaeed E.B., Taghour M.S., Badawi W.A. (2022). Design, synthesis and docking studies of new hydrazinyl-thiazole derivatives as anticancer and antimicrobial agents. J. Saudi Chem. Soc..

[54] Hassan A.A., Mohamed N.K., Aly A.A., Ramadan M., Gomaa H.A.M., Abdel-Aziz A.T., Youssif B.G.M., Bräse S., Fuhr O. (2023). Synthesis and Antiproliferative Potential of Thiazole and 4-Thiazolidinone Containing Motifs as Dual Inhibitors of EGFR and BRAFV600E. Molecules.

[55] Opitz S., Smrke S., Goodman B., Keller M., Schenker S., Yeretzian C. (2014). Antioxidant Generation during Coffee Roasting: A Comparison and Interpretation from Three Complementary Assays. Foods.

[56] Munteanu I.G., Apetrei C. (2021). Analytical Methods Used in Determining Antioxidant Activity: A Review. Int. J. Mol. Sci..

[57] Jaganjac M., Sredoja Tisma V., Zarkovic N. (2021). Short Overview of Some Assays for the Measurement of Antioxidant Activity of Natural Products and Their Relevance in Dermatology. Molecules.

[58] Wang Y., Gu D., Liu C., Tang S., Wang S., Wang Y., Yang Y. (2023). Enrichment, analysis, identification and mechanism of antioxidant components in Toona sinensis. Chin. J. Anal. Chem..

[59] Wang J., Tang H., Hou B., Zhang P., Wang Q., Zhang B.-L., Huang Y.-W., Wang Y., Xiang Z.-M., Zi C.-T. (2017). Synthesis, antioxidant activity, and density functional theory study of catechin derivatives. RSC Adv..

