# Supplementary material for: Synthesis, Cytotoxicity and Antioxidant Activity Evaluation of Some Thiazolyl–Catechol Compounds
Source: Antioxidants (Basel). 2024 Aug 1;13(8):937. doi: 10.3390/antiox13080937 (PMC11351550; doi:10.3390/antiox13080937)
Supplement: Supplementary file 1 [file antioxidants-13-00937-s001.zip › antioxidants-3104322-supplementary.pdf]

antioxidants

Supplementary Material: Synthesis, cytotoxicity and antioxidant activity of new compounds with thiazolyl-polyphenolic structure

Alexandra Cătălina Cornea [1], Gabriel Marc [1,*], Ioana Ionuț [1], Cristina Moldovan [1], Ionel Fizeșan [2,*], Andreea-Elena Petru [2], Ionuț-Valentin Creștin [2], Adrian Pîrnău [3], Laurian Vlase [4], Ovidiu Oniga [1]

1.Schemes

1.1 The synthesis route for obtention of intermediate thioamides ***1f, 1i*** *and* ***1j,*** *reported in our group previous reports*

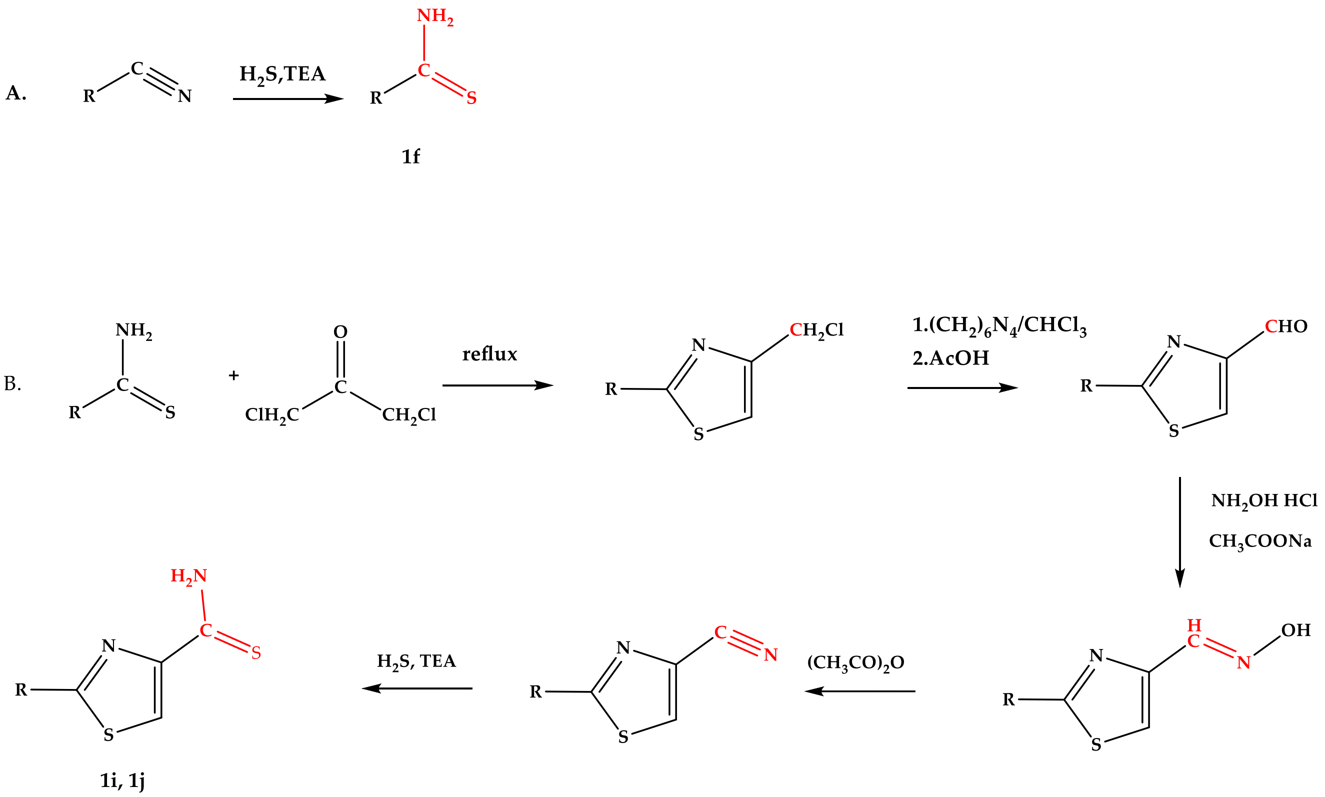

Scheme S1. Synthesis of the thioamides **1f** (A) and **1i-j** (B), according to our group previous reports [18-20]

2.Figures

2.1 The IR spectra

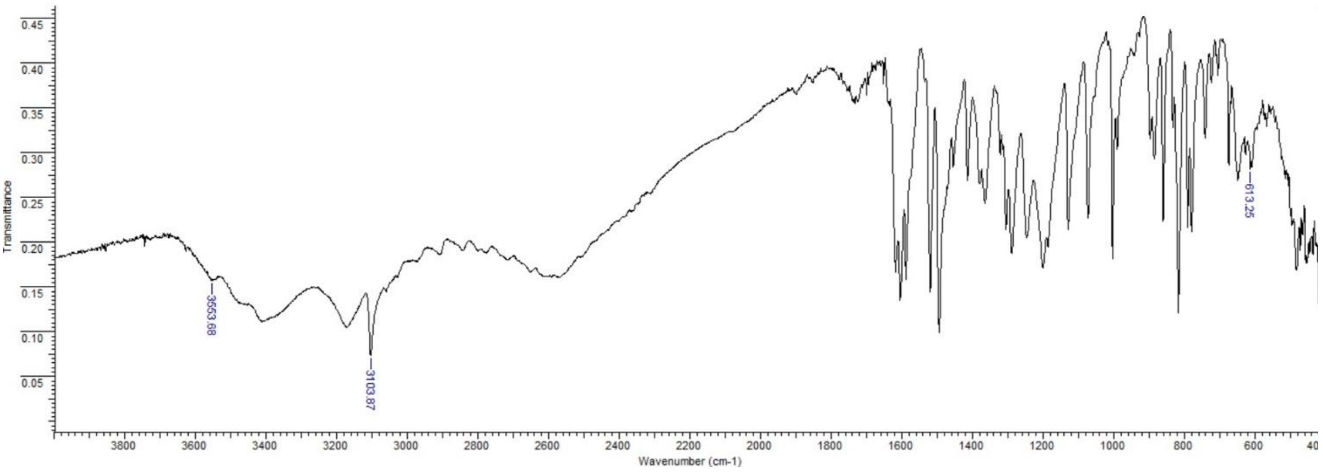

Figure S1. The IR spectrum for the compound **3d**

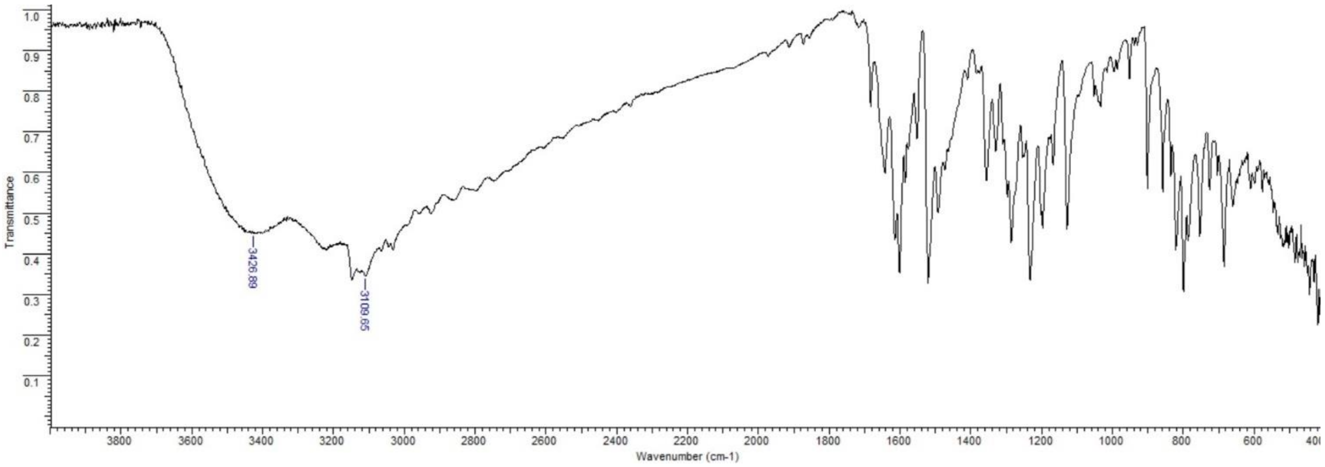

Figure S2. The IR spectrum for the compound **3e**

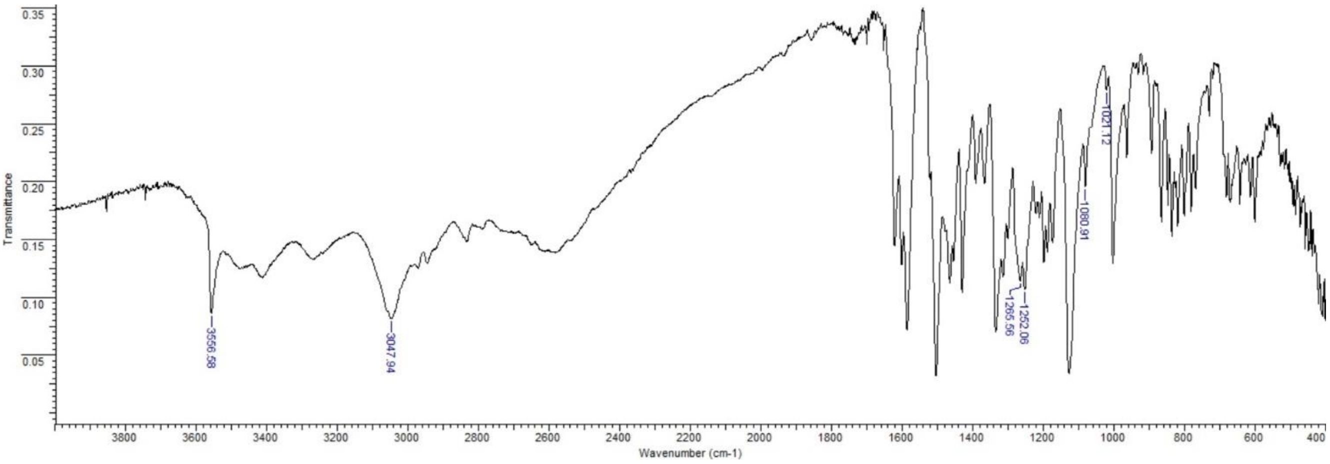

Figure S3. The IR spectrum for the compound **3f**

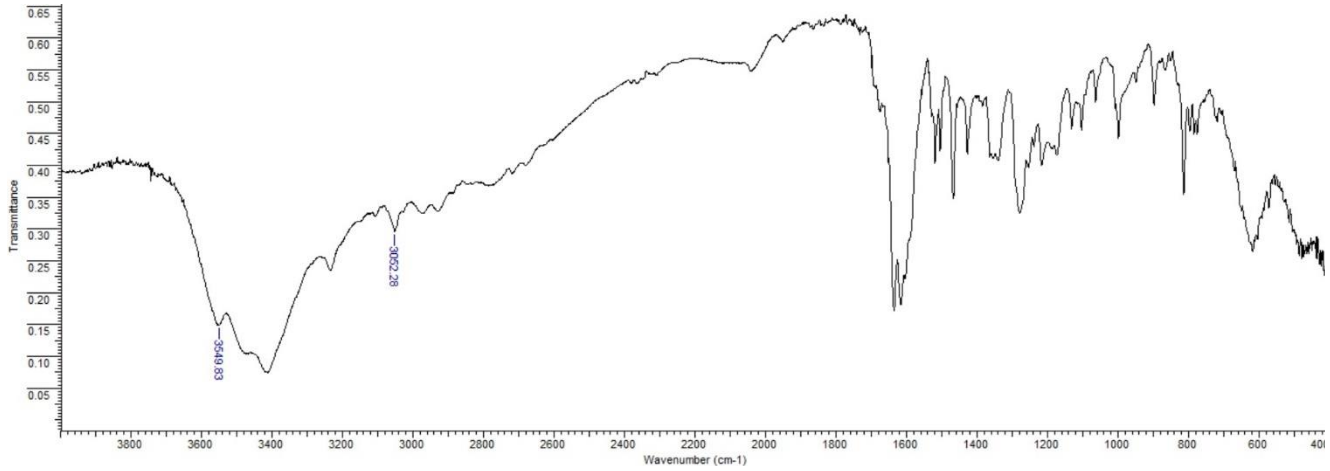

Figure S4. The IR spectrum for the compound **3g**

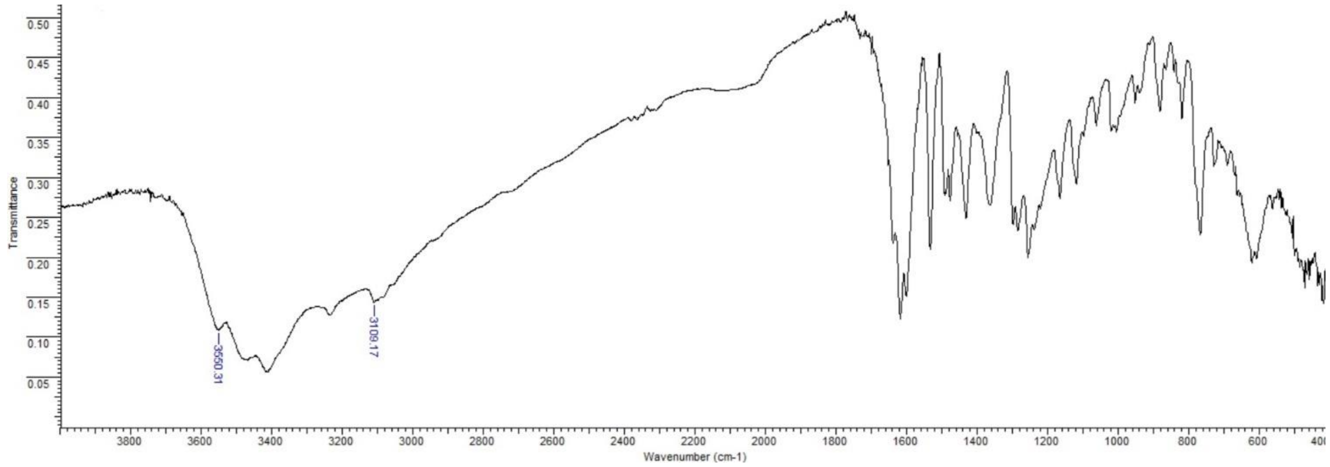

Figure S5. The IR spectrum for the compound **3h**

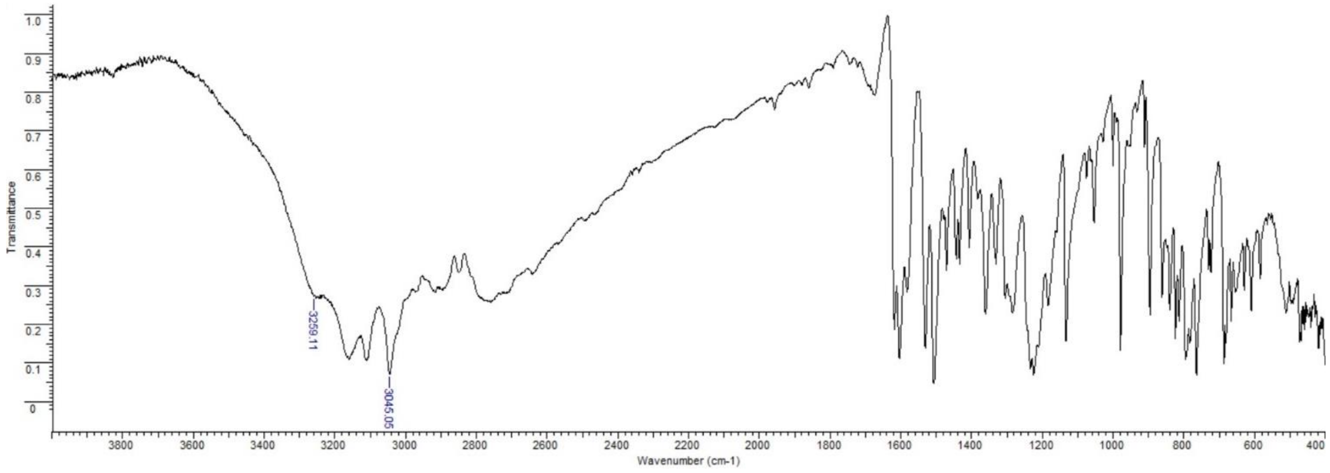

Figure S6. The IR spectrum for the compound **3i**

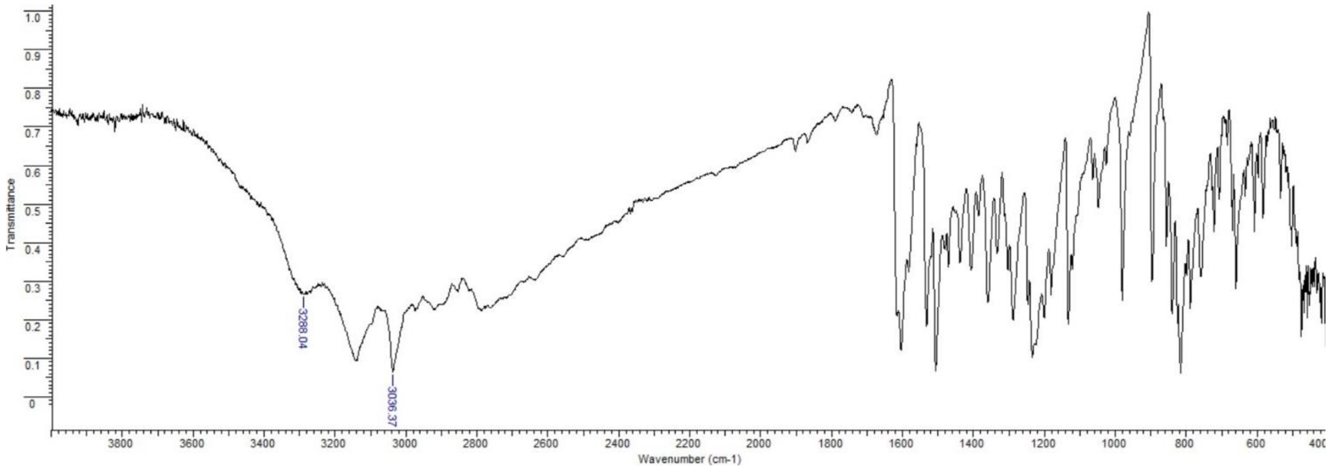

Figure S7. The IR spectrum for the compound **3j**

2.2 The ¹H-NMR spectra

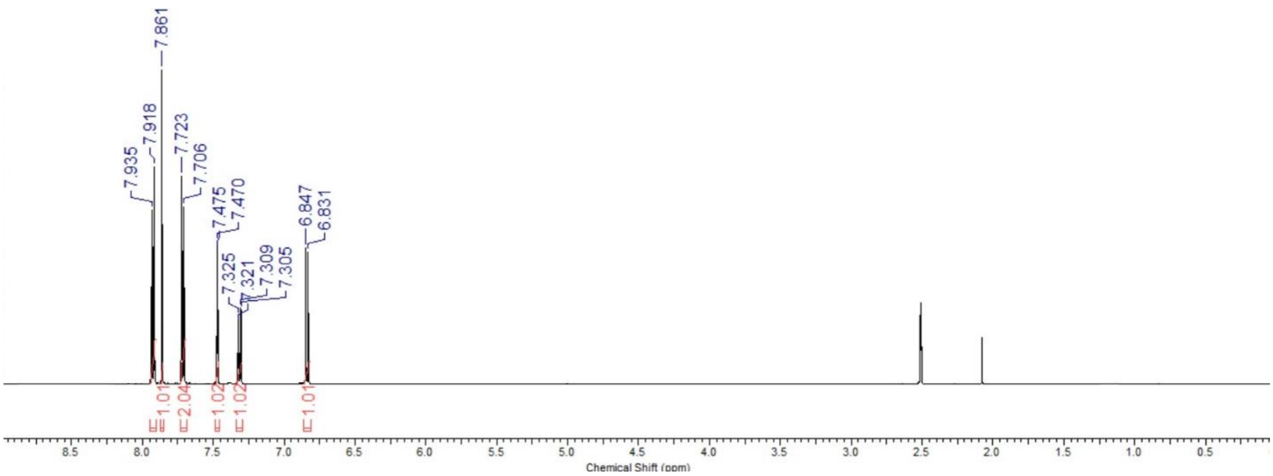

Figure S8. The 1H-NMR spectrum for the compound **3d**

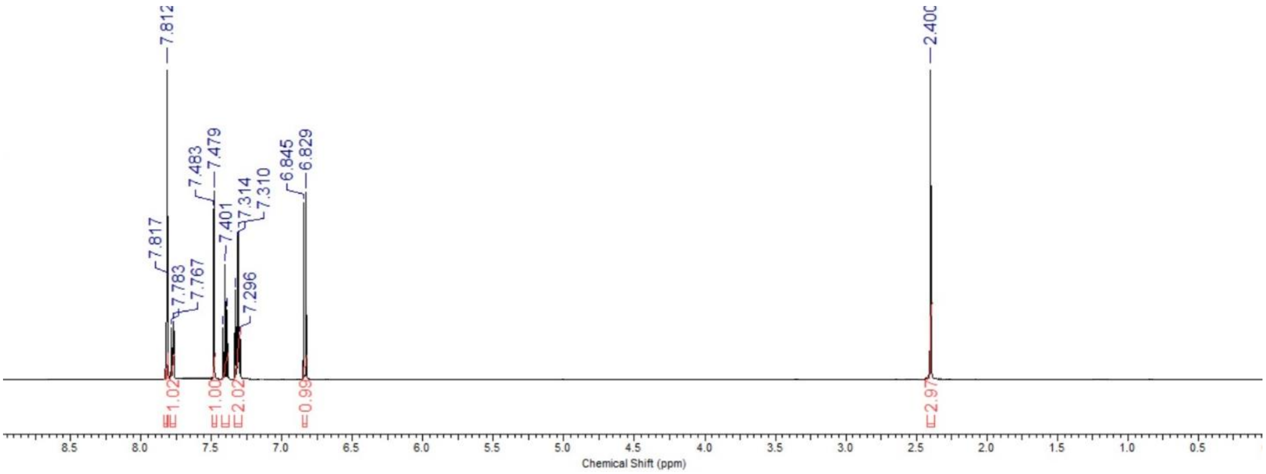

Figure S9. The 1H-NMR spectrum for the compound **3e**

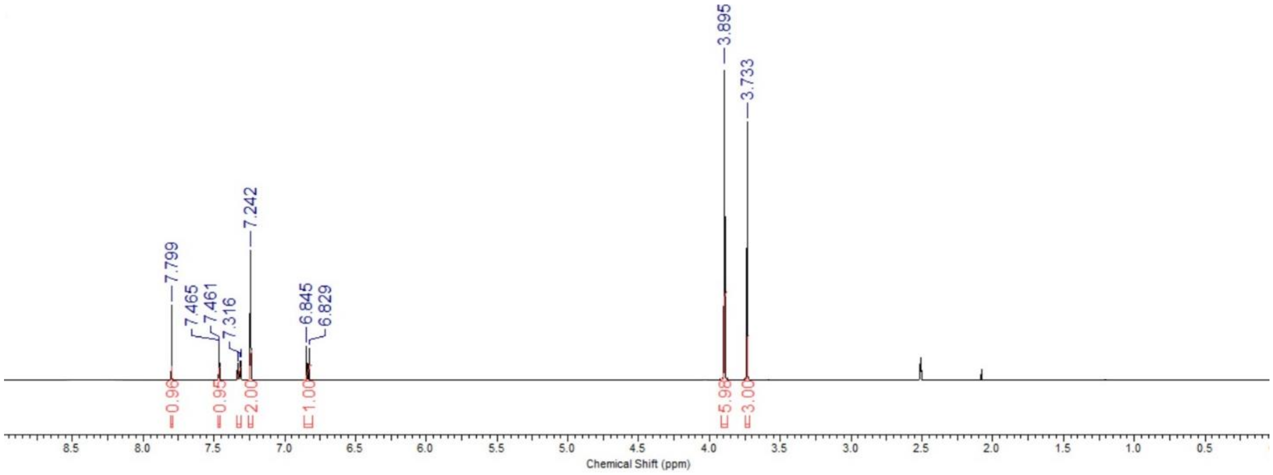

Figure S10. The 1H-NMR spectrum for the compound **3f**

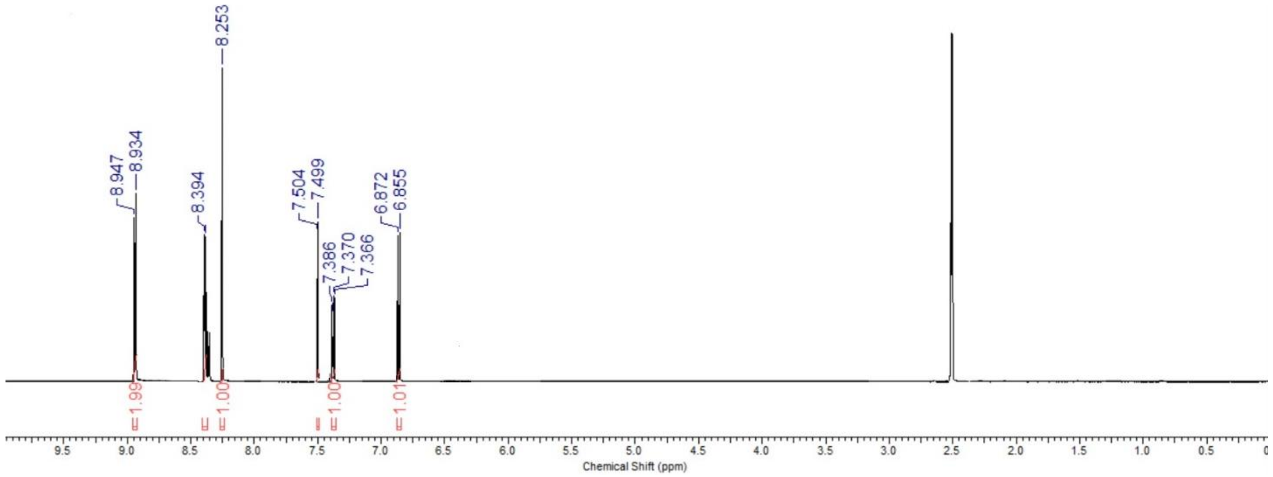

Figure S11. The 1H-NMR spectrum for the compound **3g**

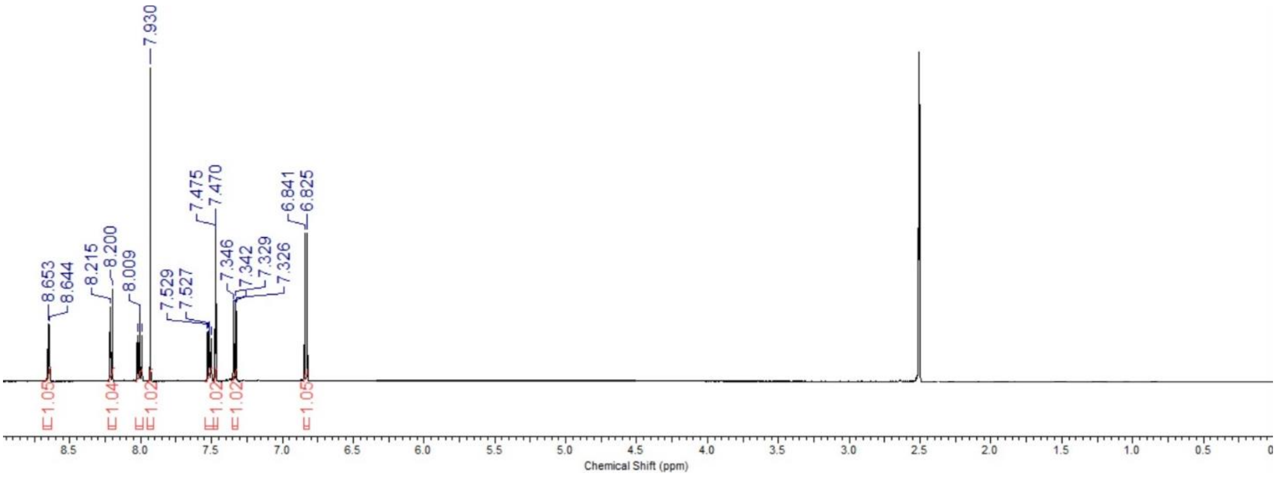

Figure S12. The 1H-NMR spectrum for the compound **3h**

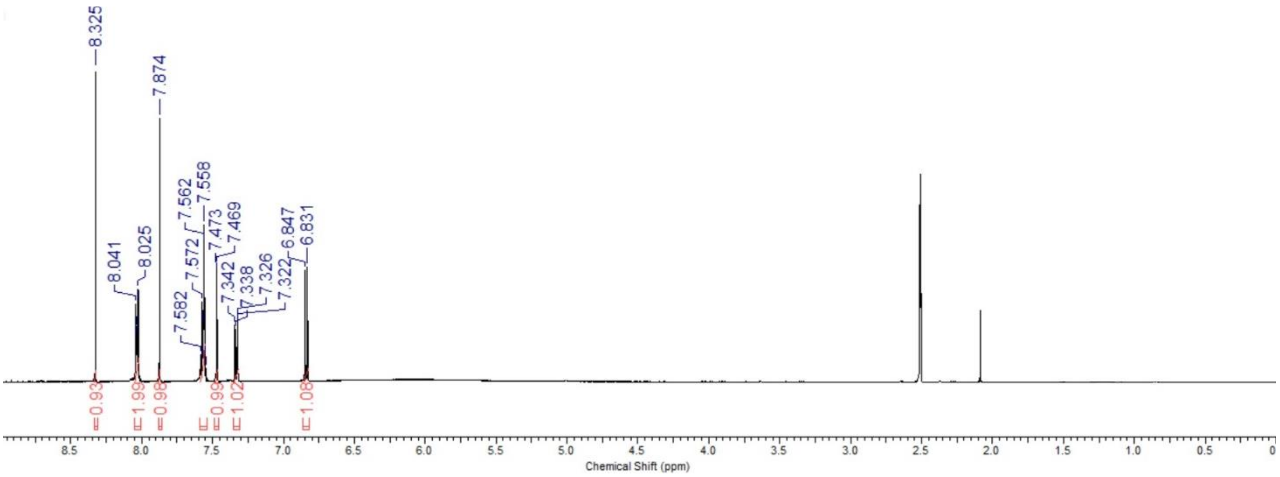

Figure S13. The 1H-NMR spectrum for the compound **3i**

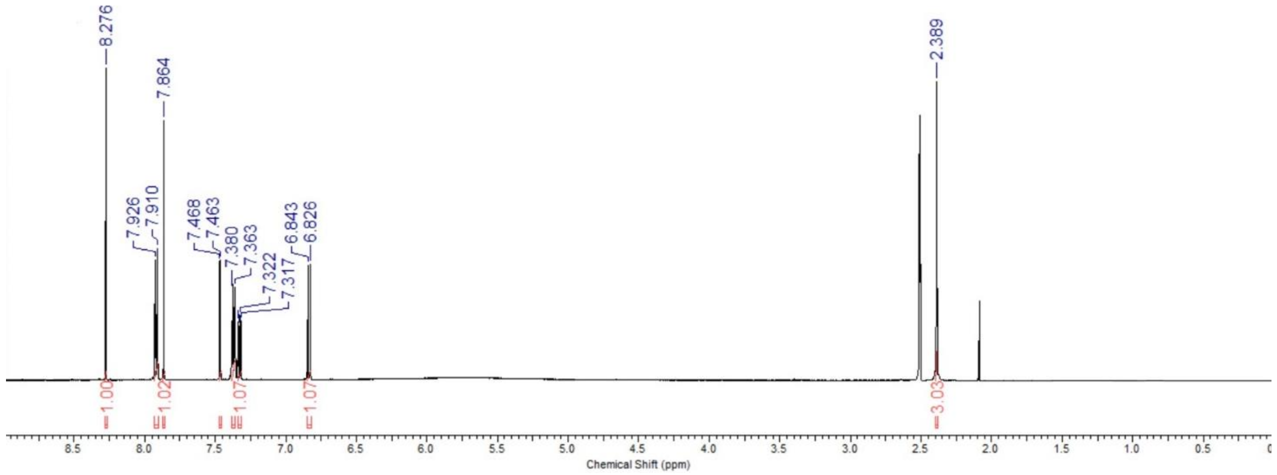

Figure S14. The 1H-NMR spectrum for the compound **3j**

2.3 The ^{13}C-NMR spectra

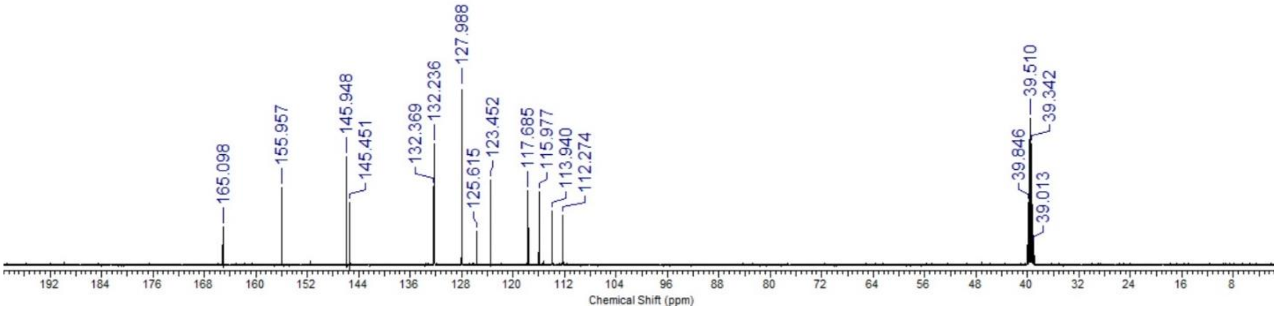

Figure S15. The ^{13}C-NMR spectrum for the compound **3d**

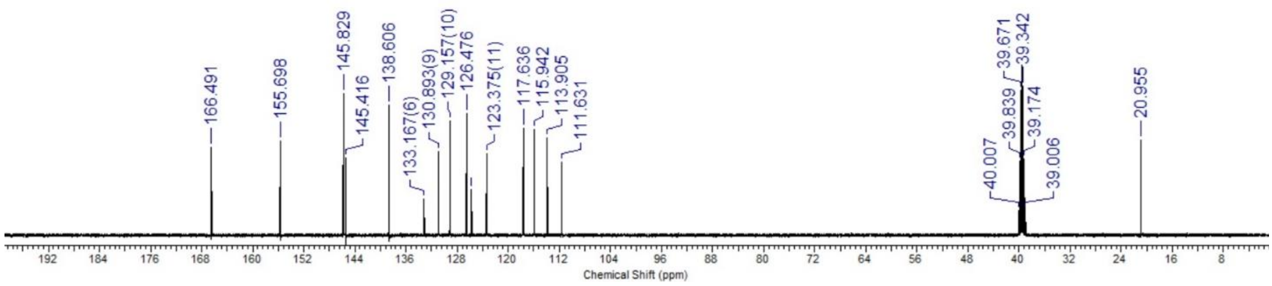

Figure S16. The ^{13}C-NMR spectrum for the compound **3e**

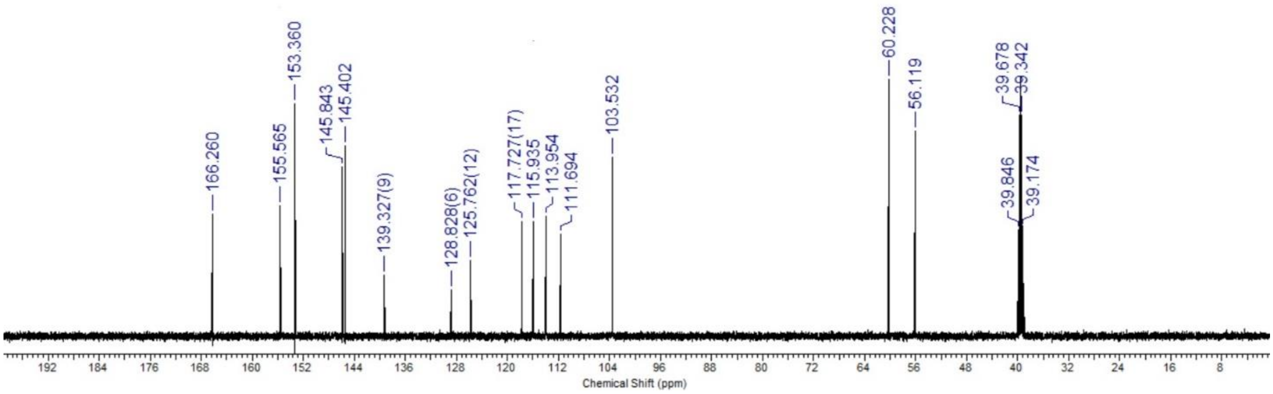

Figure S17. The ^{13}C-NMR spectrum for the compound **3f**

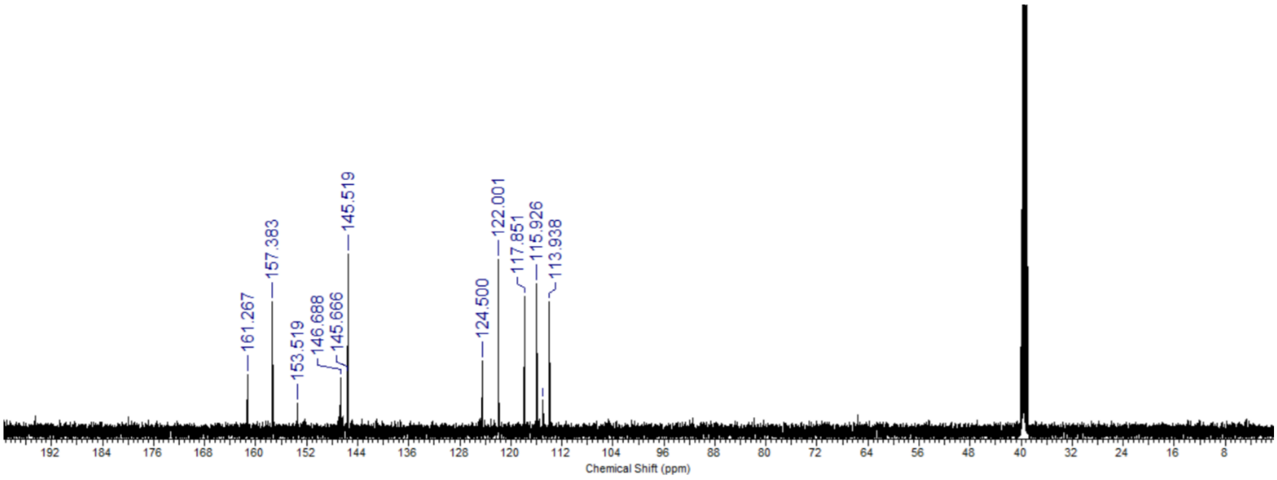

Figure S18. The ^{13}C-NMR spectrum for the compound **3g**

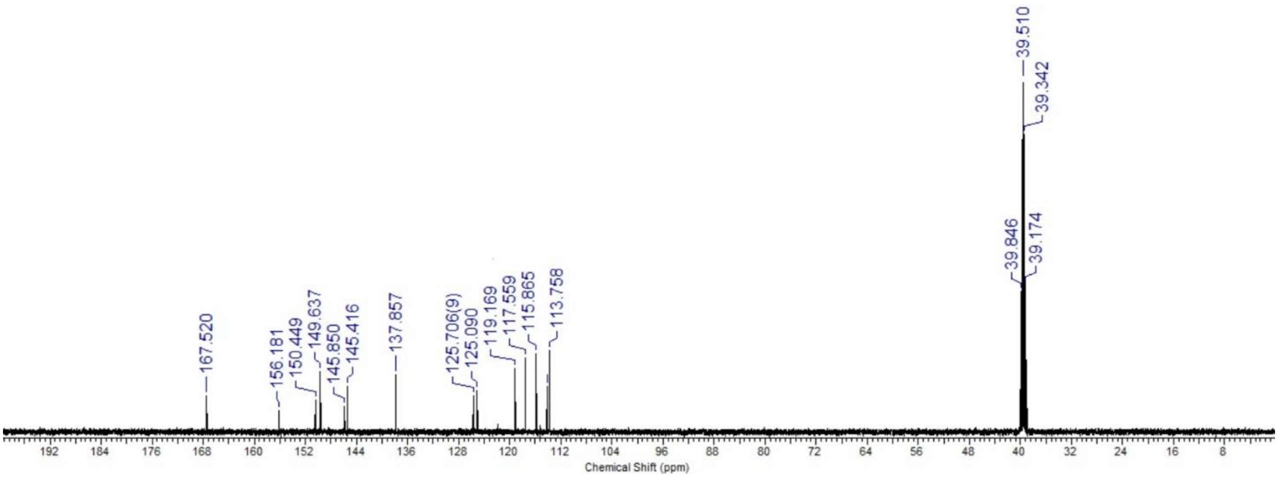

Figure S19. The ^{13}C-NMR spectrum for the compound **3h**

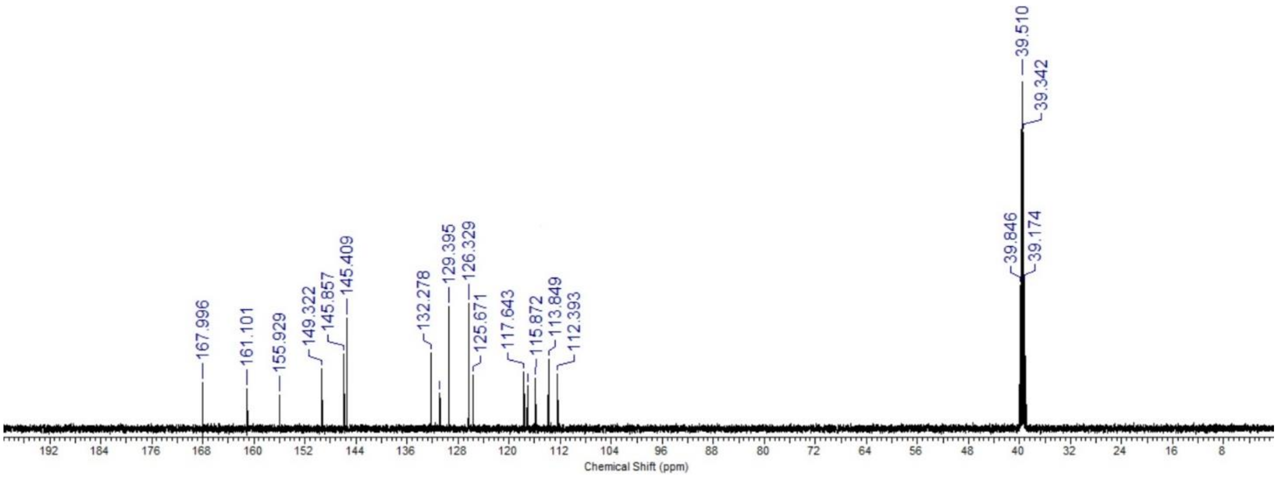

Figure S20. The ^{13}C-NMR spectrum for the compound **3i**

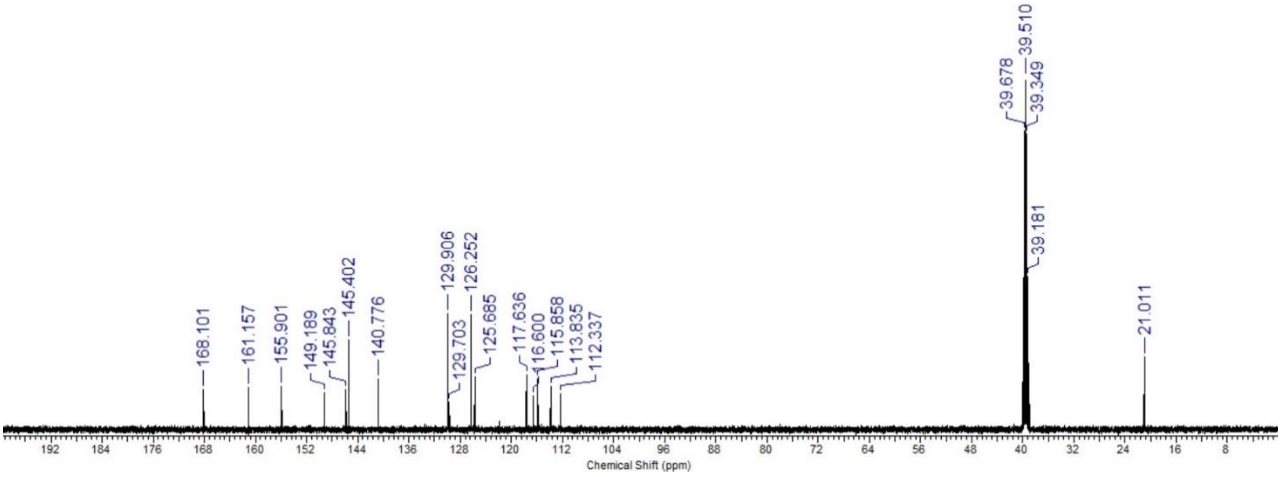

Figure S21. The ^{13}C-NMR spectrum for the compound **3j**

2.4 The MS spectra

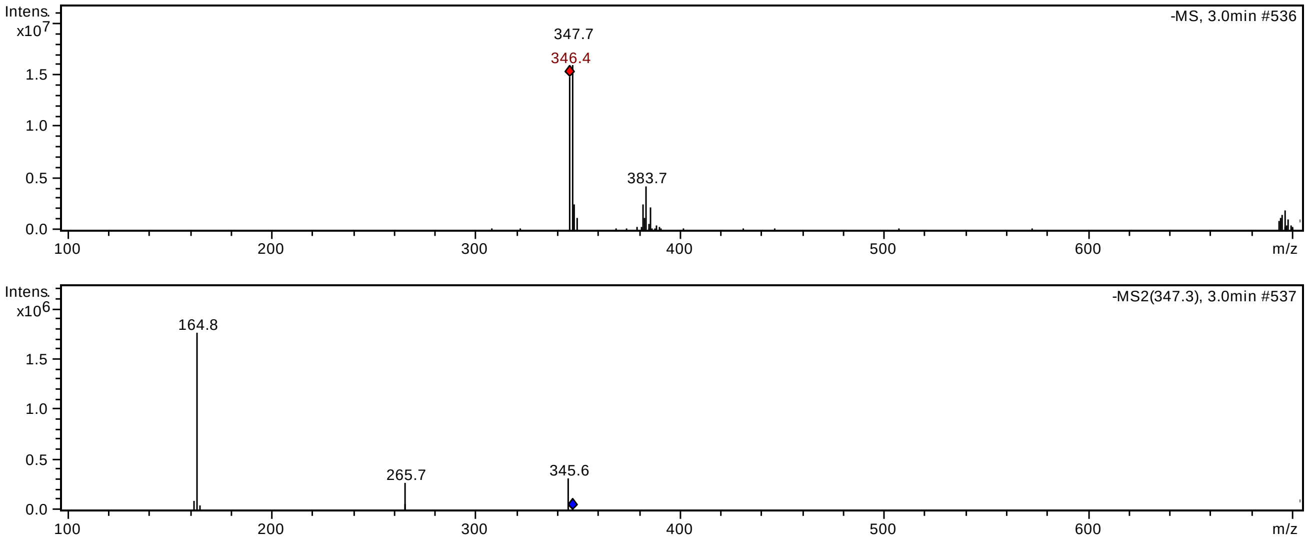

Figure S22. The MS spectrum for the compound **3d.**

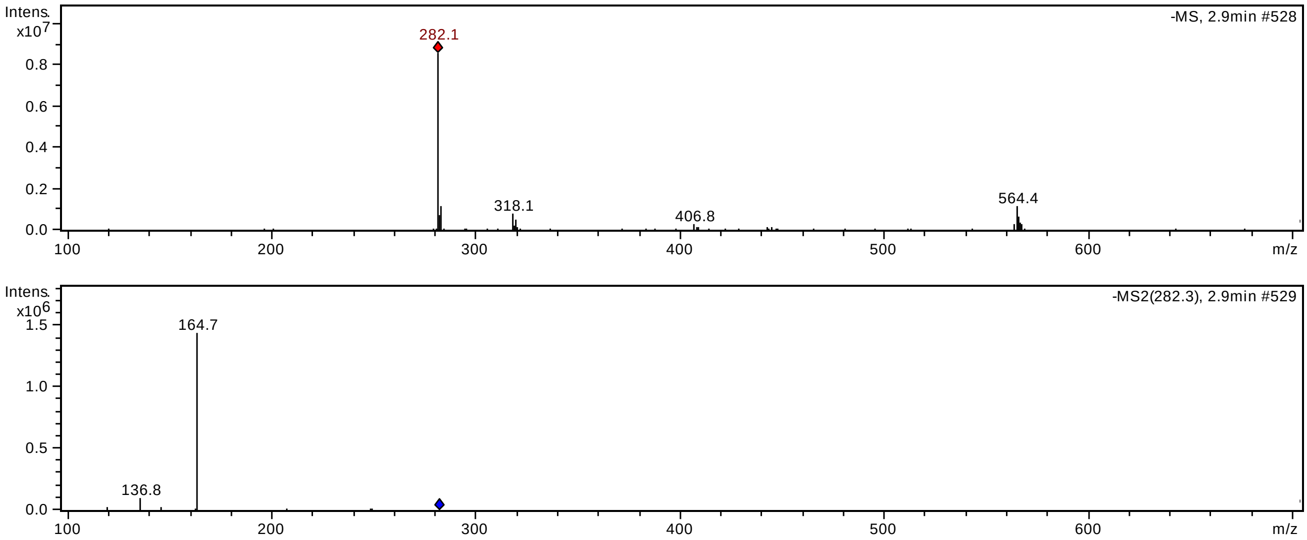

Figure S23. The MS spectrum for the compound **3e.**

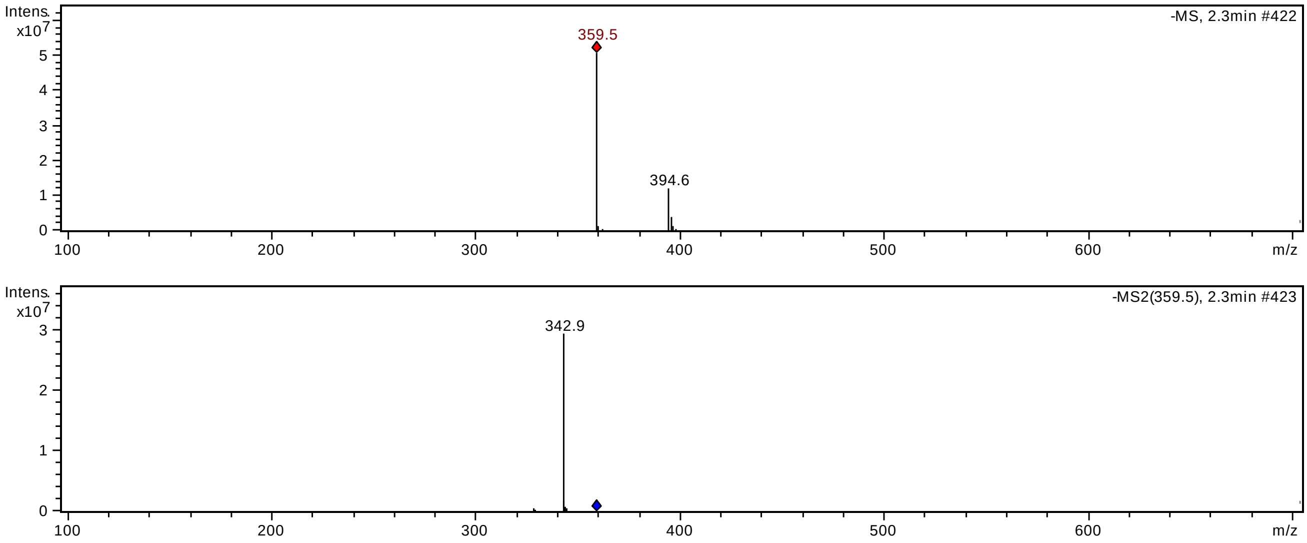

Figure S24. The MS spectrum for the compound **3f.**

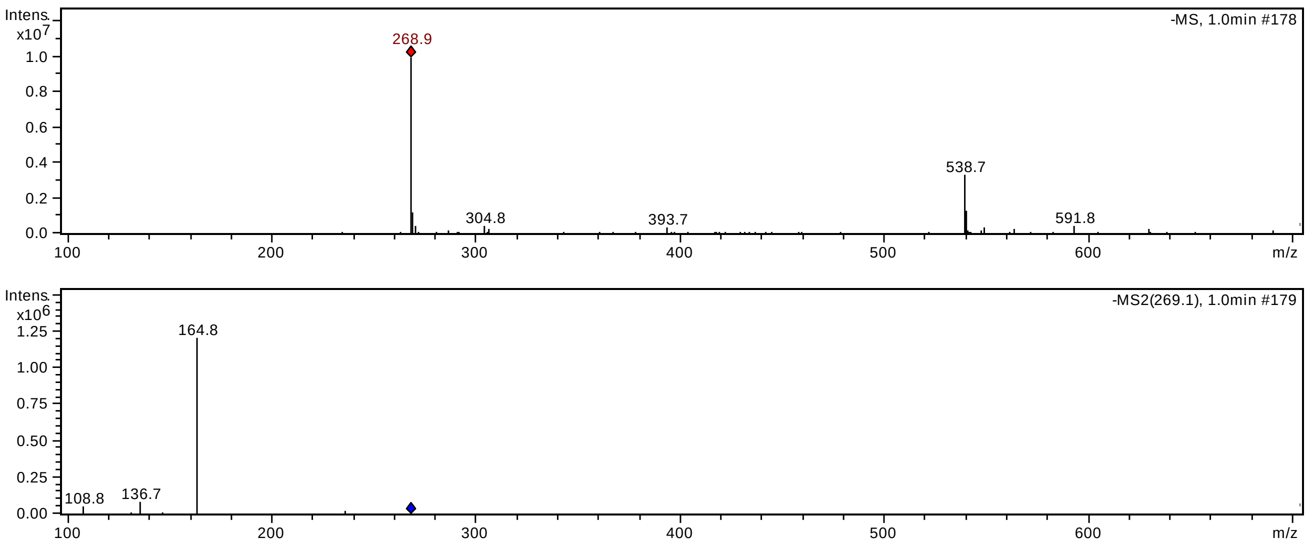

Figure S25. The MS spectrum for the compound **3g**

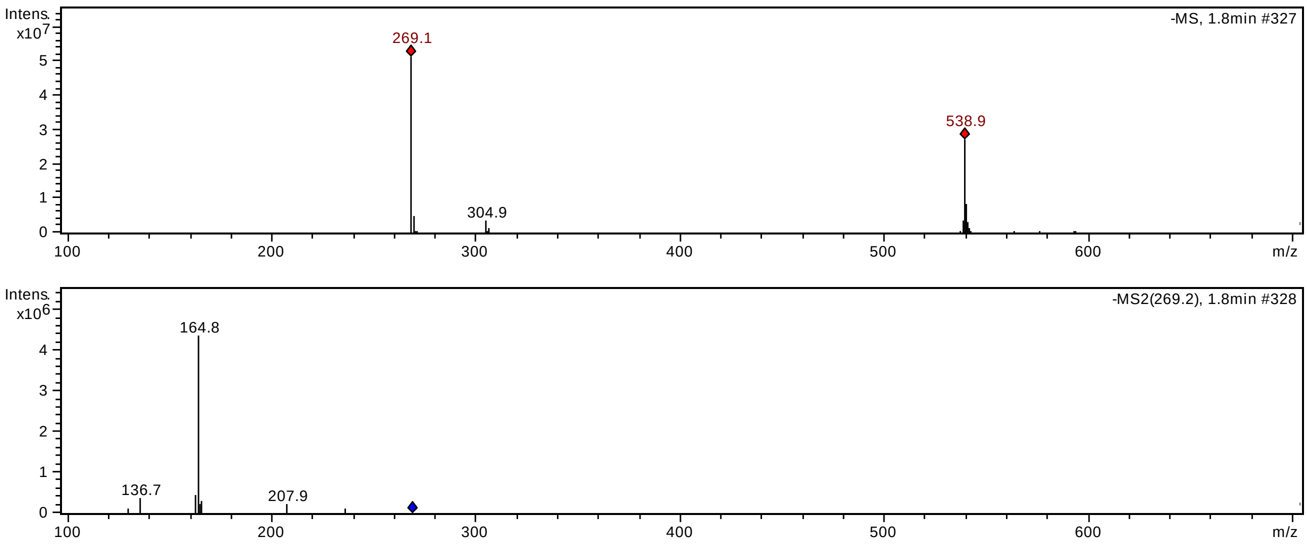

Figure S26. The MS spectrum for the compound **3h**

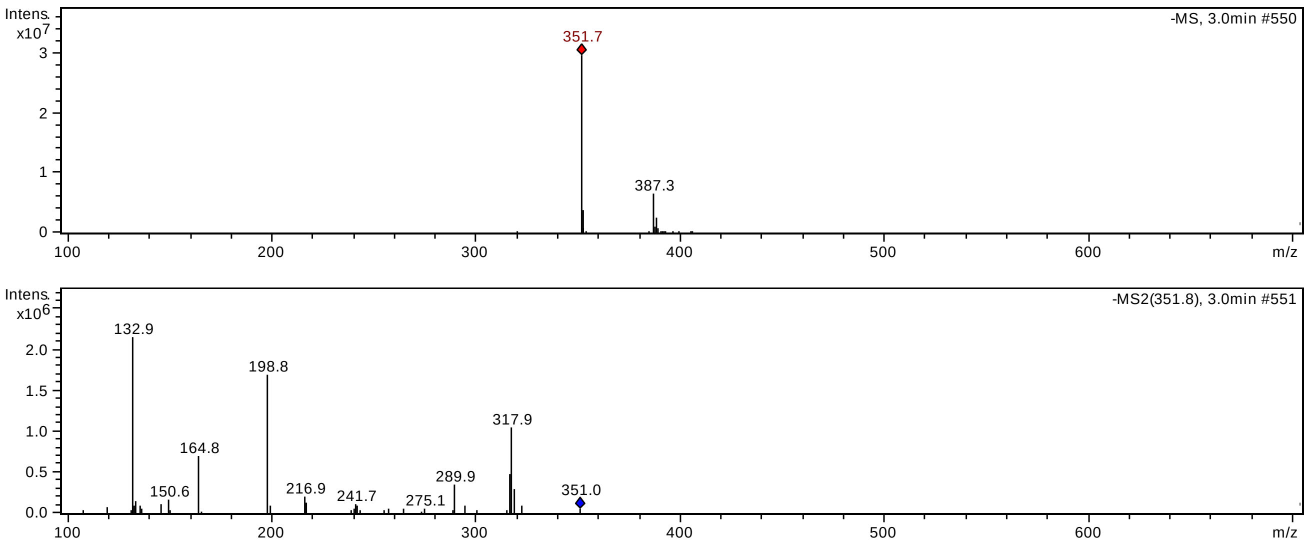

Figure S27. The MS spectrum for the compound **3i**

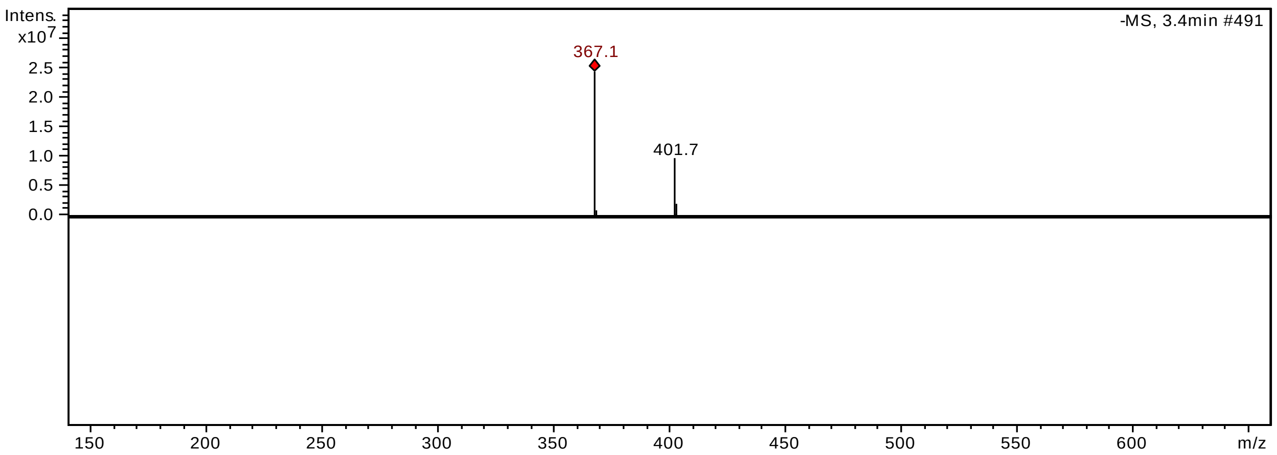

Figure S28. The MS spectrum for the compound **3j**

3. Tables

*3.1. The depiction of HOMO and LUMO and the depiction of the spin density maps of compounds **3a-j***

Table S1. The depiction of HOMO and LUMO of the compounds **3a-j**

Compound	HOMO	LUMO
**3a**	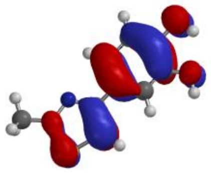	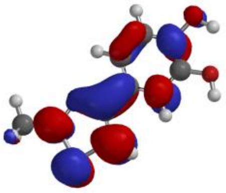
**3b**	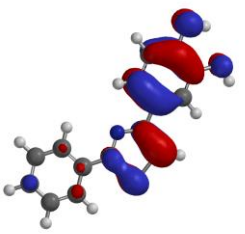	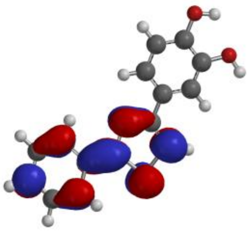
**3c**	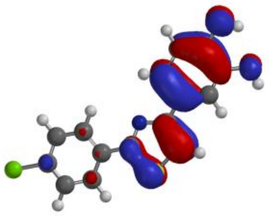	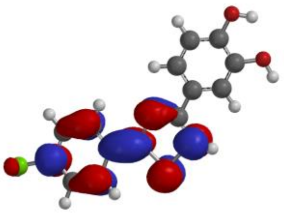

**3d**	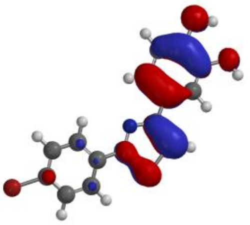	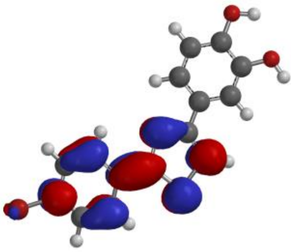
**3e**	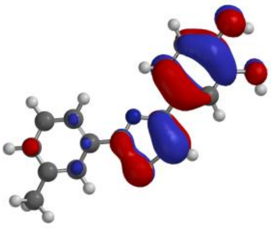	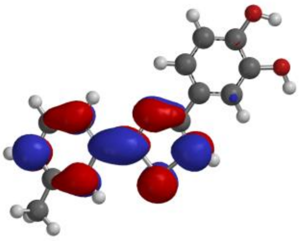
**3f**	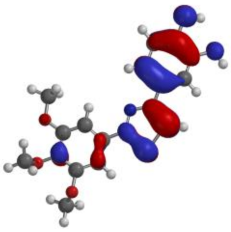	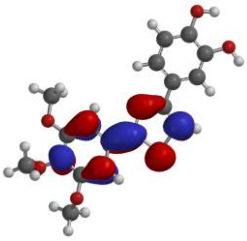
**3g**	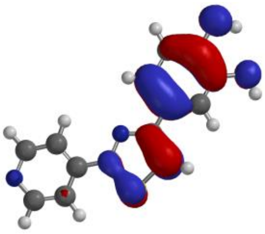	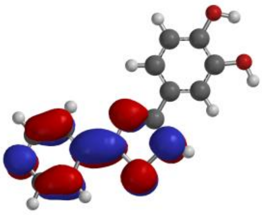
**3h**	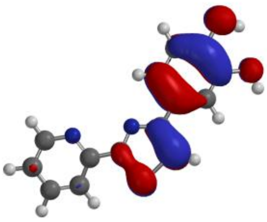	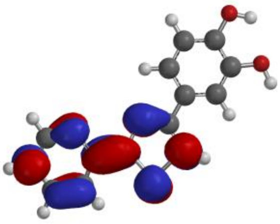
**3i**	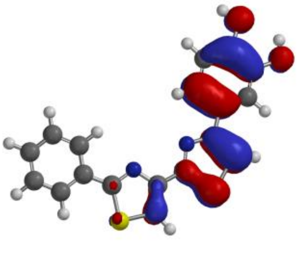	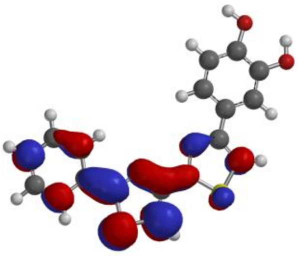

**3j**	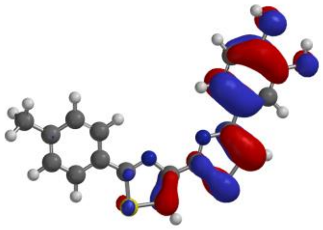	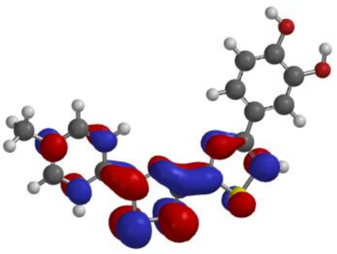

Table S2. The electrostatic potential maps of the compounds **3a-j**

**Compound**	**EPM**
**3a**	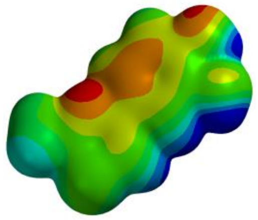
**3b**	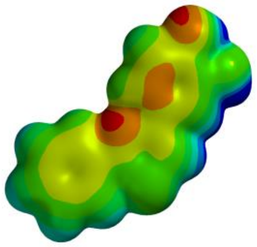
**3c**	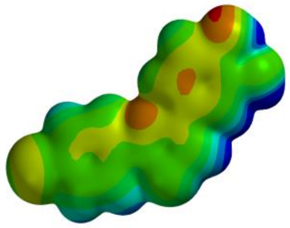
**3d**	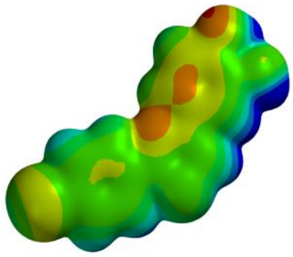

**3e**	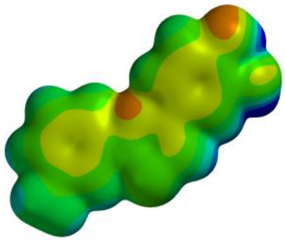
**3f**	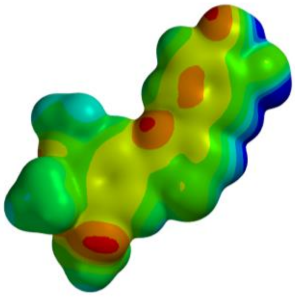
**3g**	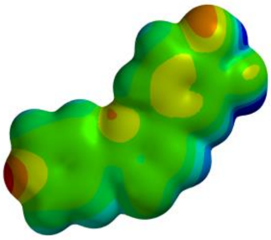
**3h**	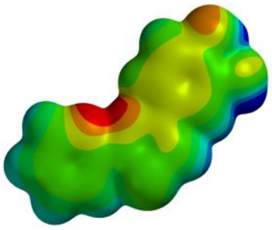
**3i**	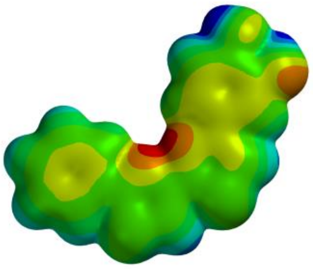
**3j**	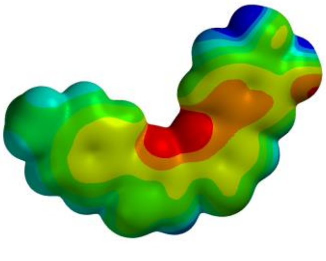

*3.2. Thermodynamic data computed for compounds **3a-j***

Table S3. Thermodynamic data computed for compounds **3a-j** in vacuum (T=298.15K, P=1atm)

Compound	ZPE (kJ/mol)	S^0 (J/mol*K)	H^0 (au)	G^0 (au)	Cv (J/mol*K)
**3a**	430.48	430.48	-989.67923	-989.737123	212.70
**3b**	563.64	563.64	-1181.36434	-1181.429950	271.52
**3c**	539.60	539.60	-1640.96879	-1641.036220	287.48
**3d**	538.18	538.18	-3754.65427	-3754.722470	288.95
**3e**	632.32	632.32	-1220.65350	-1220.723420	289.16
**3f**	806.99	806.99	-1524.81570	-1524.899090	390.98
**3g**	534.26	534.26	-1197.41177	-1197.476640	226.56
**3h**	534.03	534.03	-1197.40757	-1197.482550	226.76
**3i**	652.13	652.13	-1749.18372	-1749.259180	341.99

Table S4. Thermodynamic data computed for compounds **3a-j** in nonpolar solvent (T=298.15K, P=1atm)

Compound	ZPE (kJ/mol)	S^0 (J/mol*K)	H^0 (au)	G^0 (au)	Cv (J/mol*K)
**3a**	430.14	440.16	-989.69427	-989.75210	212.69
**3b**	563.56	487.32	-1181.38043	-1181.44601	271.42
**3c**	539.82	506.61	-1640.98501	-1641.05229	287.10
**3d**	537.86	514.85	-3754.67074	-3754.73895	289.09
**3e**	632.18	512.77	-1220.67588	-1220.73960	288.98
**3f**	807.57	602.21	-1524.83600	-1524.91916	390.39
**3g**	534.24	485.69	-1197.42978	-1197.49464	266.47
**3h**	534.19	485.05	-1197.43010	1197.49977	266.59
**3i**	652.63	550.82	-1749.21055	-1749.27902	341.51

Table S5. Thermodynamic data computed for compounds **3a-j** in water (T=298.15K, P=1atm)

Compound	ZPE (kJ/mol)	S^0 (J/mol*K)	H^0 (au)	G^0 (au)	Cv (J/mol*K)
**3a**	430.07	440.03	-989.69680	-989.75461	212.66
**3b**	563.56	487.09	-1181.38310	-1181.44870	271.34
**3c**	539.80	506.44	-1640.98775	-1641.05501	287.04
**3d**	537.79	514.86	-3754.67350	-3754.74170	289.10
**3e**	633.06	512.25	-1220.67390	-1220.74195	296.87
**3f**	807.55	602.01	-1524.83941	-1524.92254	390.36
**3g**	534.25	485.47	-1197.43282	-1197.49765	266.40
**3h**	534.18	484.99	-1197.43505	-1197.50271	266.57
**3i**	652.69	550.71	-1749.21126	-1749.28236	341.41
